# L-Ornithine-L-aspartate enhances growth performance and nitrogen metabolism via modulation of intestinal amino acid transporters and microbiota in broilers

**DOI:** 10.1186/s40104-026-01450-7

**Published:** 2026-07-03

**Authors:** Xiaodan Zhang, Guangzhi Ma, Jinping Wang, Bin Wang, Zengpeng Lv, Yuming Guo

**Affiliations:** 1 State Key Laboratory of Animal Nutrition and Feeding, College of Animal Science and Technology, China Agricultural University, Beijing, 100193 China; 2 Anhui Wanhe Jial Biotechnology Co., Ltd., Anhui, 239000 China

**Keywords:** AA-targeted metabolomics, Broilers, L-Ornithine-L-aspartate, Microbiota, Multi-omics analysis, Nitrogen metabolism

## Abstract

**Background:**

L-Ornithine-L-aspartate (OA) is a stable salt formed by the ionic bonding of ornithine and aspartic acid. While OA is known to regulate nitrogen metabolism and ammonia (NH_3_) detoxification more effectively than its individual components in clinical settings, their specific effects and mechanisms in broiler chickens remain unexplored. Crucially, it is unknown whether OA exerts superior biological effects compared to a physical mixture of ornithine and aspartic acid in broiler chickens. Therefore, this study selected white-feathered broilers to investigate the comparative effects of dietary supplementation with OA versus a mixture of ornithine and aspartic acid on growth performance, nitrogen metabolism, and intestinal health. The objective of this study was to elucidate the mechanisms underlying the potential superiority of the salt form (OA) over the mixture.

**Results:**

Compared to the basal diet, both supplementation groups improved growth performance and slaughter performance by promoting glutamine (Gln) synthesis and NH_3_ detoxification, thereby enhancing protein deposition. Specifically, supplementation significantly reduced the feed conversion ratio from d 1 to 21 and increased body weight and breast muscle percentage at d 42 (*P* < 0.05). Notably, OA demonstrated superior efficacy compared to the mixture. Mechanistically, OA significantly increased hepatic ornithine aminotransferase (OAT) activity at d 21, facilitating ornithine transamination for Gln synthesis. In the gut, OA uniquely reduced duodenal crypt depth (CD) and up-regulated the mRNA expression of key amino acid (AA) transporters (*SLC1A5*, *SLC25A15*, and *SLC38A2*) in the jejunum, leading to significantly higher apparent ileal digestibility of AAs (*P* < 0.05). Metabolomic and microbiomic analyses revealed that, compared to the mixture, OA significantly modulated arginine biosynthesis pathways (gga00220) and down-regulated L-ornithine (C00077) abundance in ileal chyme. Furthermore, OA improved the cecal microbiota by increasing the relative abundance of butyrate-producing *Agathobaculum*, reducing pathogenic *Escherichia*, and up-regulating energy metabolism pathways.

**Conclusions:**

In summary, while both forms are beneficial, OA is superior to the physical mixture of ornithine and aspartic acid in improving broiler performance. This advantage of the OA was correlated with the up-regulated intestinal AA transporters, improved the digestibility of nutrients such as ornithine, and the modulated gut microbiota towards a butyrate-producing profile, coupled with increased hepatic OAT activity.

**Graphical Abstract:**

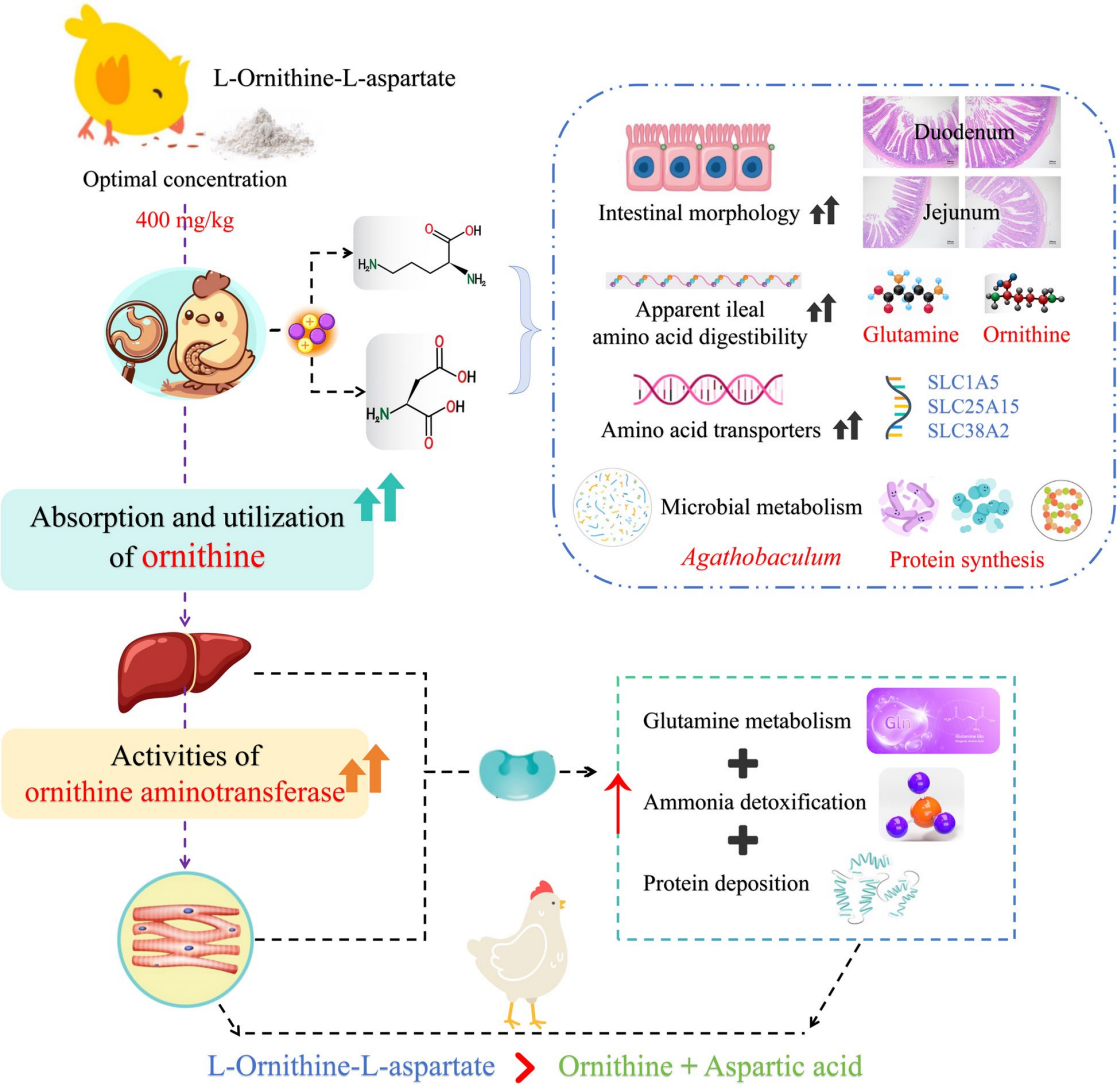

**Supplementary Information:**

The online version contains supplementary material available at 10.1186/s40104-026-01450-7.

## Background

Nitrogen metabolism is a pivotal determinant of growth performance in broilers. Under physiological conditions, dietary proteins and amino acids (AAs) are digested and metabolized, a process that inevitably yields ammonia (NH_3_) as a toxic by-product. Given its cytotoxicity, NH_3_ must be efficiently transported to the liver, the central hub for nitrogen regulation and detoxification [1]. Within hepatic tissue, the interconversion between glutamate (Glu) and glutamine (Gln) plays a critical role in NH_3_ detoxification metabolism while simultaneously modulating protein synthesis and deposition [2–4]. Furthermore, hepatic AA metabolism, involving transamination and deamination, generates essential precursors for various biosynthetic pathways and regulates signaling networks governing protein metabolism and tissue development [5]. Specifically, ornithine and aspartic acid serve as key substrates in transamination reactions to produce Glu, thereby promoting Gln synthesis and facilitating NH_3_ clearance. This metabolic cascade directly impacts the growth performance, muscle development, and overall health of broiler chickens [6]. Additionally, gut health remains a prerequisite for the efficient digestion and absorption of nitrogenous nutrients such as protein and AAs. Consequently, the strategic regulation of hepatic nitrogen metabolism, coupled with the maintenance of gut health, is essential for optimizing nutrient utilization, enhancing growth performance, and increasing farming profitability in broiler production [7].

L-Ornithine-L-aspartate (OA) is a stable salt complex comprising ornithine and aspartic acid linked by ionic bonds [8], thereby combining the functional properties of both AAs. Upon dietary ingestion, OA readily dissociates into its constituent AAs, ornithine and aspartic acid, within the acidic environment of the upper gastrointestinal tract. Subsequently, these AAs are absorbed and utilized in the intestine via active transport mechanisms [8]. Following dissociation, ornithine and aspartic acid are transported across the apical membrane of intestinal epithelial cells by specific AA transporters, such as ORNT1 and ASCT2. Once intracellular, they not only serve as metabolic substrates but also function as signaling molecules, interacting with intracellular AA sensors [9, 10]. It is well-established that following absorption into the systemic circulation, these metabolites are distributed to peripheral tissues, including the liver and muscle tissue, for metabolism and utilization. This bioavailability is a fundamental prerequisite for OA to exert its physiological effects. Numerous studies have demonstrated that OA enhances Gln metabolism by stimulating Gln synthesis and promoting protein deposition, which in turn facilitates muscle development. Additionally, it mitigates the production of NH_3_ and uric acid (UA), effectively alleviating pathologies such as sarcopenia and hepatic dysfunction associated with hyperammonemia [11–13]. Similar to these findings, our previous research indicated that OA supplementation effectively lowers blood ammonia levels by modulating nitrogen metabolism, enhances protein deposition in both hepatic and muscle tissues, and significantly improves the growth performance of broiler chickens.

Compared to individual AAs, AA complex salts formed via ionic bonding through acid–base neutralization not only retain the individual effects of their constituents but may also confer additional advantages, such as synergistic metabolic effects. Specifically, OA is hypothesized to possess superior bioavailability and intestinal absorption rates compared to its individual counterparts [14]. Numerous studies have demonstrated that OA exhibits therapeutic efficacy superior to that of separate ornithine or aspartic acid supplementation [15–17]. However, comparative studies evaluating the specific effects of OA versus a simple mixture of ornithine and aspartic acid in broiler chickens are currently lacking. Consequently, whether OA exerts superior biological effects compared to an equivalent mixture in broilers, and the mechanisms driving such potential advantages, remain to be elucidated. Therefore, the present study was designed to investigate and compare the effects of dietary supplementation with OA versus an ornithine-aspartic acid mixture on the growth performance, slaughter performance, and nitrogen metabolism of one-day-old Arbor Acres broilers fed corn-soybean meal (SBM)-based diets. The objective was to reveal the differential effects of these forms and to uncover the potential mechanisms underlying the advantageous role of OA.

## Materials and methods

### Experimental animals, diets, and management

The study was performed at the Poultry Breeding Base of Zhuozhou Teaching and Experimental Farm, China Agricultural University (Hebei, China). The feeding management and nutritional supply of the broilers were strictly maintained in accordance with the recommendations and regulations of the Feeding Management Manual.

In Exp. 1, a total of 210 one-day-old male Arbor Acres broilers were randomly divided into three dietary treatments, with five replicates of 14 chickens each. The dietary treatments were as follows: (1) negative control (NC): corn-SBM-based basal diet; (2) positive control (PC): basal diet supplemented with 200 mg/kg L-ornithine and 200 mg/kg L-aspartic acid; and (3) experimental group (OA): basal diet supplemented with 400 mg/kg OA. This specific level was selected based on our laboratory’s extensive preliminary dose–response studies and repeated validation trials, which consistently identified 400 mg/kg as the optimal inclusion level for maximizing growth performance and regulating nitrogen metabolism in broilers.

The basal diets were formulated to meet the nutritional requirements for broilers as recommended by the Chicken Feeding Standard of China (NY/T33–2004) [18]. All experimental diets were formulated to be isonitrogenous and isoenergetic. The ingredient composition and nutrient levels of the basal diets are presented in Table 1. In Exp. 2, 210 one-day-old Arbor Acres broilers were used. The experimental design and treatment grouping were identical to Exp. 1. However, titanium dioxide (TiO_2_) was added to the diets as an exogenous indigestible marker to determine nutrient digestibility (Table 2).

**Table 1:** Ingredients and composition of the basal diet in Exp. 1^a^ (as is basis)

Ingredients	Percentage, %	
	d 1 to 21	d 22 to 42
Corn (7.8% crude protein (CP))	45.68	50.02
SBM (44% CP)	26.81	25.54
Corn gluten meal	9.70	5.39
SBM oil	3.38	5.32
Flours	10.00	10.00
L-Lysine hydrochloride (98.5%)	0.53	0.27
Calcium hydrogen phosphate	1.91	1.33
Stone powder	0.91	1.14
Choline chloride (50%)	0.20	0.16
DL-Methionine (99%)	0.15	0.10
Antioxidants	0.03	0.03
NaCl	0.35	0.35
Vitamin premix^b^	0.03	0.03
Trace mineral feed^c^	0.20	0.20
Phytase	0.02	0.02
alanine	0.10	0.10
Calculated nutrient levels^d^		
Metabolizable energy, Mcal/kg	3.14	3.21
CP	23.93	20.79
Ca	0.97	0.89
Total phosphorus	0.74	0.62
Available phosphorus	0.41	0.31
Lysine	1.28	1.03
Methionine	0.52	0.41
Methionine + Cystine	0.85	0.70

^a^The feed is in pellet form

^b^The analytical values per kg of vitamin premix composition are as follows: Vitamin A 50 million IU, Vitamin D_3_ 12 million IU, Vitamin E 100,000 IU, Vitamin K_3_ 10 g, Vitamin B_1_ 8 g, Vitamin B_2_ 32 g, Vitamin B_6_ 12 g, Vitamin B_12_ 100 mg, Nicotinamide 150 g, D-pantothenic acid 46 g, Folic acid 5 g, Biotin 500 mg, moisture ≤ 6%

^c^The analytical values per kg of trace mineral feed ingredients are as follows: Cu 8, Fe 40, Zn 55, Mn 60 g, I 750, Se 150, Co 250 mg, moisture ≤ 10%

^d^Values calculated from the analysis of the test diets

**Table 2:** Ingredients and composition of the basal diet in Exp. 2^a^ (as is basis)

Ingredients	Percentage, %	
	d 1 to 21	d 22 to 42
Corn (7.8% CP)	45.43	49.64
SBM (44% CP)	26.68	25.41
Corn gluten meal	9.88	5.52
SBM oil	3.36	5.29
Flours	9.73	9.93
L-Lysine hydrochloride (98.5%)	0.53	0.27
Calcium hydrogen phosphate	1.90	1.33
Stone powder	0.91	1.13
Choline chloride (50%)	0.20	0.15
DL-Methionine (99%)	0.15	0.10
Antioxidants	0.03	0.03
NaCl	0.35	0.35
Vitamin premix^b^	0.03	0.03
Trace mineral feed^c^	0.20	0.20
Phytase	0.02	0.02
alanine	0.10	0.10
TiO_2_	0.50	0.50
Calculated nutrient levels^d^		
Metabolizable energy, Mcal/kg	3.13	3.20
CP	23.93	20.77
Ca	0.97	0.89
Total phosphorus	0.74	0.62
Available phosphorus	0.41	0.31
Lysine	1.28	1.03
Methionine	0.52	0.41
Methionine + Cystine	0.85	0.70

^a^The feed is in pellet form

^b^The analytical values per kg of vitamin premix composition are as follows: Vitamin A 50 million IU, Vitamin D_3_ 12 million IU, Vitamin E 100,000 IU, Vitamin K_3_ 10 g, Vitamin B_1_ 8 g, Vitamin B_2_ 32 g, Vitamin B_6_ 12 g, Vitamin B_12_ 100 mg, Nicotinamide 150 g, D-pantothenic acid 46 g, Folic acid 5 g, Biotin 500 mg, moisture ≤ 6%

^c^The analytical values per kg of trace mineral feed ingredients are as follows: Cu 8, Fe 40, Zn 55, Mn 60 g, I 750, Se 150, Co 250 mg, moisture ≤ 10%

^d^Values calculated from the analysis of the test diets

### Growth performance

On d 21 and 42, broilers were weighed on a per-cage basis following a 12-h fasting period. Feed consumption was recorded concurrently for each replicate. Based on these measurements, average feed intake (AFI), average gain (AG), and feed conversion rate (FCR) were calculated for d 1–21, d 22–42, and d 1–42, respectively. Additionally, the general health status and condition of the flock were monitored daily throughout the experiment.

### Slaughter performance

In Exp. 1, two broilers were randomly selected from each replicate at 42 days of age. Following individual weighing, they were euthanized via jugular exsanguination. Slaughter performance traits were evaluated in strict accordance with the performance terminology and measurement guidelines for poultry (NY/T 823–2020) [19].

### Sample collection and preparation

On d 21 and 42, one broiler was randomly selected from each replicate and weighed. Blood samples were collected from the wing vein, followed by intravenous injection of sodium pentobarbital at a dose of 30 mg/kg body weight. Subsequently, broilers were euthanized via jugular exsanguination. Serum was separated by centrifugation at 3,000 × *g* for 15 min at 4 °C. Liver and breast muscle tissues were rapidly harvested and flash-frozen in liquid nitrogen for molecular analysis. In Exp. 2, additional sampling was conducted on d 21. One broiler per replicate was selected, and tissue samples (approximately 1.5 cm) of the mid-duodenum and mid-jejunum were collected. These segments were gently rinsed with physiological saline and fixed in 4% paraformaldehyde for intestinal morphology analysis. Concurrently, jejunal and cecal chyme were aseptically collected and snap-frozen in liquid nitrogen. All sera, tissues, and samples were stored at −80 °C until analysis.

### Serum biochemical analysis

Serum biochemical parameters were analyzed using an automated biochemical analyzer (Chemray 800, Shenzhen Leidu, China). The measured parameters included the activities of alanine aminotransferase (ALT) and aspartate aminotransferase (AST), as well as the concentrations of UA, blood urea nitrogen (BUN), albumin (ALB), globulin (GLB), and total protein (TP). All assays were performed by Wuhan Servicebio Technology Co., Ltd. (Wuhan, China).

### Nitrogen metabolism

#### Nitrogen metabolism-related enzyme activities and metabolite concentrations

The activities of glutamine synthase (GS, AKAM008M), glutaminase (GLS, AKAM007M), and xanthine oxidase (XOD, AKAO006M) were determined in both serum and breast muscle samples using commercial assay kits (Beijing Boxbio Science & Technology Co., Ltd., Beijing, China). Hypoxanthine phosphoribosyltransferase (HPRT1, YX-150101C) activity was measured in serum samples using a specific assay kit (Shanghai Guduo Biotechnology Co., Ltd., Shanghai, China). Additionally, the activity of ornithine aminotransferase (OAT) in liver tissue samples was assessed using a specific ELISA kit (AKAM013U). The assay was performed strictly in accordance with the manufacturer’s instructions.

The concentrations of NH_3_ (AKBL009M) and Gln (AKAM027M) in both serum and breast muscle were determined using commercial assay kits purchased from Beijing Boxbio Science & Technology Co., Ltd. For the assessment of growth-related factors, chicken-specific ELISA kits were employed. Specifically, the levels of insulin-like growth factor 1 (IGF-1) and myostatin (GDF-8), as well as serum growth hormone (GH), were quantified using kits purchased from Shanghai Guduo Biotechnology Co., Ltd. (Shanghai, China). All biochemical assays were performed in strict accordance with the manufacturers’ protocols.

#### Expression analysis of nitrogen metabolism-related genes and pathways

Total RNA was isolated from liver and breast muscle tissues using TRIzol reagent. The concentration and purity of the extracted RNA were assessed using a NanoDrop 2000 microspectrophotometer (Thermo Fisher Scientific, Waltham, MA, USA). Quantitative real-time PCR was performed to evaluate the expression levels of genes related to nitrogen metabolism, with β-actin serving as the endogenous control. The specific primer sequences used in this study are listed in Table S1. Relative gene expression levels were calculated using the 2^−ΔΔCt^ method.

### Intestinal histology

Tissue samples from duodenum and jejunum were embedded in paraffin and sectioned. The sections were stained with hematoxylin–eosin for histological evaluation. Images were captured and analyzed using a microscope (DMI8, Leica, Wetzlar, Germany). For each sample, ten intact, straight-running villi were randomly selected for measurement. Villi height (VH) and crypt depth (CD) were quantified using LIOO software, and the VH to CD ratio (V/C) was calculated.

### Apparent ileal AA digestibility (AIAAD)

Ileal chyme samples were collected from the distal half of the ileum. The collected chyme was immediately frozen at −20 °C. Subsequently, samples were freeze-dried and ground for analysis. The AA contents of both the diet and the ileal chyme samples were determined in accordance with the National Standard GB/T 18246–2019. TiO_2_ was used as an exogenous indicator, and its concentration in the feed and chyme was analyzed using the formula.

### Targeted metabolomics analysis of AAs

Ileal chyme samples collected on d 21 were subjected to targeted AA metabolomics analysis using liquid chromatography-tandem mass spectrometry (LC–MS/MS). Standard curves for each AA were constructed using the multiple reaction monitoring mode, and absolute quantification was performed in conjunction with stable isotope-labeled internal standards. To ensure data reliability, quality control (QC) samples were evaluated. Spectral overlap comparisons of total ion chromatograms (TIC) were performed, and Pearson correlation analysis on the QC samples was also conducted. Principal component analysis (PCA) was used to outline the population structure, and orthogonal partial least squares discriminant analysis (OPLS-DA) was used to analyze differences between treatments. The aforementioned testing was conducted by Shanghai Personal Biotechnology Co., Ltd. (Shanghai, China).

### Expression analysis of intestinal AA transporter-related genes

TRIzol reagent was used to extract total RNA from jejunum samples, and the relative mRNA expression levels of intestinal AA transporter-related genes were determined (Table S2).

### 16S rRNA sequencing of cecal microbiota

Microbial DNA was extracted from cecal chyme samples using the QIAamp Fast DNA Stool Mini Kit (QIAGEN, Germany) following the manufacturer’s instructions. DNA concentration was quantified using a NanoDrop 2000 microspectrophotometer, while DNA purity was assessed via 1% agarose gel electrophoresis. The common primers 515 F (5′-GTG CCA GCM GCC GCG GTA A-3′) and 806R (5′-GGA CTA CHV GGG TWT CTA AT-3′) targeting the V4 region of the 16S rRNA gene were used to amplify the bacterial DNA. Based on the concentration of PCR products, equal amounts of samples were mixed. PCR products were detected by 2% agarose gel electrophoresis, and recovery of the products was performed from the target bands using the Gel Extraction Kit from QIAGEN.

Paired-end reads were merged using Flash software (version 1.2.7) to obtain raw tags. These raw tags were subsequently subjected to quality filtering following the QIIME (version 1.9.1) quality control protocol. Chimeric sequences were identified and removed using the UCHIME algorithm to ensure data integrity. Taxonomic classification was performed at various hierarchical levels based on ASV/OTU counts. Data visualization, including the generation of heatmaps and bar charts, was conducted using igraph (version 2.0.3) and ggplot2 (version 3.5.1). To identify differentially abundant taxa, Student’s *t*-tests were performed for genera with a relative abundance greater than 0.001 (0.1%). PICRUSt2 software was used to predict and analyze metagenomic function. Furthermore, microbial association networks and community structure analyses were performed to elucidate ecological interactions.

### Concentrations of short-chain fatty acids (SCFAs) in cecal chyme

The concentrations of SCFAs in cecal chyme were analyzed using a gas chromatograph (SHIMADZU GC2014) following the method described in our previous study [7]. Quantification was performed using the internal standard method.

### Correlation analysis

A correlation analysis was performed to evaluate the associations among the measured parameters. This analysis aimed to elucidate the interrelationships between key indicators, thereby providing insights into the potential mechanisms by which OA exerts its advantageous effects on broiler performance and metabolism. Correlation analysis was completed using the Wekemo Bioincloud.

### Statistical analysis

SPSS 22.0 software was used for statistical analysis of the data from each treatment group. One-way analysis of variance (ANOVA) was used for statistical analysis of the data. The differences between treatments were analyzed by Duncan’s multiple range test, and the results were expressed as mean ± standard error of the mean (SEM). All statements of significant differences were based on *P* < 0.05 and the *P* values between 0.05 and 0.1 were classified as trends.

## Results

### Exp. 1

#### Growth performance

The results of broiler growth performance are shown in Table 3. Compared with the control group fed the basal diet, the group supplemented with 400 mg/kg OA had significantly increased BW at d 21 and AG from d 1 to 21 (*P* < 0.05). Furthermore, OA supplementation significantly improved feed efficiency, as evidenced by a reduced FCR from d 22 to 42 and from d 1 to 42 (*P* < 0.05). Compared with the negative control, both the OA group and the PC group showed significantly decreased FCR from d 1 to 21, increased BW at d 42, and enhanced AG from d 1 to 42 (*P* < 0.01). Notably, the 400 mg/kg OA group exhibited superior efficacy in improving growth performance compared to the mixture group. In conclusion, OA supplementation proved to be more effective than the simple mixture of its constituent AAs in promoting broiler growth.

**Table 3:** Effects of dietary supplementation with an ornithine-aspartic acid mixture and OA on growth performance of broiler chickens (*n* = 5)

Item	NC	PC	OA	*P*-value
d 1 to 21				
BW, kg	0.920^b^ ± 0.010	0.945^ab^ ±0.006	0.959^a^ ± 0.009	0.027
AG, kg	0.875^b^ ± 0.010	0.899^ab^ ±0.007	0.914^a^ ± 0.009	0.027
AFI, kg	1.084 ± 0.009	1.093 ± 0.005	1.085 ± 0.008	0.669
FCR	1.240^a^ ± 0.008	1.215^b^ ± 0.006	1.188^c^ ± 0.009	0.002
d 22 to 42				
BW, kg	2.959^b^ ± 0.018	3.035^a^ ± 0.017	3.072^a^ ± 0.017	0.002
AG, kg	2.038^b^ ± 0.023	2.090^ab^ ±0.023	2.112^a^ ± 0.012	0.058
AFI, kg	2.992 ± 0.044	3.030 ± 0.052	2.965 ± 0.055	0.667
FCR	1.468^a^ ± 0.008	1.449^ab^ ±0.010	1.403^b^ ± 0.024	0.039
d 1 to 42				
AG, kg	2.913^b^ ± 0.019	2.989^a^ ± 0.017	3.026^a^ ± 0.017	0.002
AFI, kg	4.076 ± 0.043	4.123 ± 0.048	4.050 ± 0.062	0.607
FCR	1.399^a^ ± 0.008	1.379^a^ ± 0.009	1.338^b^ ± 0.017	0.011

^a,b ^The data in the same row with shoulder labels containing different letters indicate significant differences (*P* < 0.05)

#### Slaughter performance

As shown in Table 4, compared with the negative control group, dietary supplementation with OA or the ornithine-aspartic acid mixture significantly increased breast muscle percentage (BMP) in 42-day-old broilers (*P* < 0.05). These findings indicate that both treatments effectively enhanced slaughter performance at d 42, specifically by promoting breast muscle accretion.

**Table 4:** Effects of dietary supplementation with an ornithine-aspartic acid mixture and OA on slaughter performance of broiler chickens at d 42 (*n* = 10), %

Item	NC	PC	OA	*P*-value
Dressing percentage (DP)	93.323 ± 0.250	94.484 ± 0.570	94.261 ± 0.442	0.161
Half-eviscerated percentage (HEP)	88.834 ± 0.132	89.514 ± 0.942	88.494 ± 0.575	0.527
Eviscerated percentage (EP)	77.163 ± 0.245	77.567 ± 0.946	77.062 ± 0.553	0.845
BMP	30.323^b^ ± 0.600	31.943^a^ ± 0.415	32.008^a^ ± 0.433	0.035
Leg muscle percentage (LMP)	20.220 ± 0.395	21.228 ± 0.392	21.018 ± 0.593	0.293
Abdominal fat percentage (AFP)	1.727 ± 0.065	1.552 ± 0.090	1.512 ± 0.097	0.185

^a,b^ The data in the same row with shoulder labels containing different letters indicate significant differences (*P* < 0.05)

#### Serum biochemical analysis

As presented in Table 5, dietary supplementation with 400 mg/kg OA elicited significant improvements in serum biochemical parameters. Specifically, compared to the negative control group, the OA-supplemented group exhibited a significant reduction in serum ALT activity at d 21 (*P* < 0.01), as well as decreased AST activity and serum UA levels at d 42 (*P* < 0.05). Furthermore, dietary supplementation with OA or the ornithine-aspartic acid mixture significantly increased serum GLB and TP levels at d 21 (*P* < 0.01), while concurrently reducing UA concentrations (*P* < 0.05). Additionally, supplementation with the ornithine-aspartic acid mixture significantly reduced serum BUN levels at d 42 (*P* < 0.05) compared to the negative control.

**Table 5:** Effects of dietary supplementation with an ornithine-aspartic acid mixture and OA on blood biochemical parameters in broiler chickens (*n* = 5)

Item	NC	PC	OA	*P*-value
d 21				
ALT, U/L	2.792^a^ ± 0.075	2.589^ab^ ±0.081	2.321^b^ ± 0.104	0.009
AST, U/L	397.759^a^ ± 33.269	330.432^ab^ ±13.936	310.072^b^ ± 14.032	0.041
UA, μmol/L	705.495^a^ ± 38.596	556.264^b^ ± 58.517	554.893^b^ ± 25.659	0.046
BUN, mg/dL	2.187 ± 0.108	1.802 ± 0.237	1.902 ± 0.050	0.223
ALB, g/L	13.951 ± 0.731	14.534 ± 0.529	15.510 ± 0.645	0.260
GLB, g/L	14.435^b^ ± 0.581	16.824^a^ ± 0.110	16.258^a^ ± 0.310	0.002
TP, g/L	28.386^b^ ± 0.797	31.358^a^ ± 0.608	31.768^a^ ± 0.566	0.007
d 42				
ALT, U/L	3.465 ± 0.061	2.919 ± 0.366	3.088 ± 0.117	0.252
AST, U/L	634.784 ± 75.306	501.070 ± 39.549	569.554 ± 21.873	0.217
UA, μmol/L	484.550^a^ ± 64.611	424.592^ab^ ±46.363	296.541^b^ ± 20.772	0.044
BUN, mg/dL	1.943^a^ ± 0.106	1.621^b^ ± 0.085	1.744^ab^ ±0.021	0.043
ALB, g/L	14.891 ± 0.469	15.384 ± 0.635	16.160 ± 0.726	0.374
GLB, g/L	19.492 ± 1.048	20.349 ± 0.805	21.876 ± 1.153	0.279
TP, g/L	34.384 ± 0.841	35.733 ± 0.744	38.036 ± 1.245	0.058

^a,b ^The data in the same row with shoulder labels containing different letters indicate significant differences (*P* < 0.05)

#### Nitrogen metabolism-related enzyme activities and metabolite concentrations

As presented in Table 6, compared with the NC group, dietary supplementation with 400 mg/kg OA significantly increased serum HPRT1 activity, hepatic OAT activity, and breast muscle GS activity at d 21 (*P* < 0.05). Additionally, the 400 mg/kg OA treatment significantly decreased GLS activity at d 42 (*P* < 0.05). Furthermore, all supplemented groups exhibited a significant reduction in breast muscle GLS activity at d 21 (*P* < 0.01). At d 42, both the OA group and the PC group showed significantly decreased serum XOD activity and increased serum GS activity compared to the negative control group (*P* < 0.05).

**Table 6:** Effects of dietary supplementation with an ornithine-aspartic acid mixture and OA on nitrogen metabolism-related enzyme activities in broiler chickens (*n* = 5)

Item	NC	PC	OA	*P*-value
d 21				
Serum				
GS, U/mL	1.567 ± 0.044	2.483 ± 0.394	2.339 ± 0.201	0.057
GLS, U/mL	7.493 ± 0.277	6.573 ± 0.326	6.467 ± 0.468	0.134
XOD, U/mL	0.003 ± 0.001	0.003 ± 0.001	0.002 ± 0.001	0.708
HPRT1, U/L	408.568^b^ ± 18.944	435.846^ab^ ±16.745	477.215^a^ ± 10.241	0.029
Breast muscle				
GS, U/mg prot	0.228^b^ ± 0.009	0.256^ab^ ±0.020	0.297^a^ ± 0.019	0.040
GLS, U/mg prot	0.016^a^ ± 0.003	0.010^b^ ± 0.001	0.007^b^ ± 0.001	0.006
XOD, U/mg prot	2.991 × 10^−4a^ ± 0.000	1.181 × 10^−4b^ ± 0.000	1.608 × 10^−4b^ ± 0.000	0.019
Liver				
OAT, U/mg prot	3.528^b^ ± 0.380	4.757^ab^ ±0.337	5.495^a^ ± 0.626	0.034
d 42				
Serum				
GS, U/mL	4.412^b^ ± 0.531	6.098^a^ ± 0.379	6.198^a^ ± 0.456	0.030
GLS, U/mL	7.923^a^ ± 0.555	7.356^ab^ ±0.345	6.265^b^ ± 0.336	0.048
XOD, U/mL	0.052 ± 0.014	0.030 ± 0.012	0.023 ± 0.007	0.189
HPRT1, U/L	407.609 ± 14.536	439.815 ± 14.505	448.962 ± 13.072	0.134
Breast muscle				
GS, U/mg prot	0.297 ± 0.021	0.320 ± 0.019	0.370 ± 0.019	0.061
GLS, U/mg prot	0.008 ± 0.002	0.004 ± 0.000	0.004 ± 0.001	0.051
XOD, U/mg prot	6.697 × 10^–4^ ± 0.000	6.302 × 10^–4^ ± 0.000	4.383 × 10^–4^ ± 0.000	0.826
Liver				
OAT, U/mg prot	3.343^b^ ± 0.707	5.031^ab^ ±0.287	6.230^a^ ± 0.582	0.010

^a,b^ The data in the same row with shoulder labels containing different letters indicate significant differences (*P* < 0.05)

The concentrations of nitrogen metabolism-related metabolites are presented in Table 7. Compared with the basal diet group, OA supplementation significantly elevated IGF-1 levels in both serum and breast muscle at d 21. Furthermore, at d 42, the OA group exhibited a significant reduction in breast muscle NH_3_ accumulation and blood ammonia levels, concomitant with a marked increase in Gln concentrations (*P* < 0.05). Compared with the negative control group, dietary supplementation with OA or the ornithine-aspartic acid mixture significantly reduced blood ammonia levels in 21-day-old broilers (*P* < 0.01). This was accompanied by increased Gln content and decreased GDF-8 levels in breast muscle (*P* < 0.05). Additionally, compared with both the positive and negative control groups, the addition of 400 mg/kg OA significantly increased serum GH and IGF-1 levels in broilers at d 42 (*P* < 0.05).

**Table 7:** Effects of dietary supplementation with an ornithine-aspartic acid mixture and OA on nitrogen metabolism-related metabolite concentrations in broiler chickens (*n* = 5)

Item	NC	PC	OA	*P*-value
d 21				
Serum				
Gln, nmol/mL	303.740^b^ ± 32.894	468.487^a^ ± 72.278	496.811^a^ ± 40.724	0.044
NH_3_, μmol/mL	0.964^a^ ± 0.007	0.789^b^ ± 0.005	0.793^b^ ± 0.004	< 0.001
IGF-1, ng/mL	15.590^b^ ± 0.980	18.179^ab^ ±1.003	19.410^a^ ± 0.982	0.050
GH, ng/mL	2.556 ± 0.051	2.842 ± 0.101	2.979 ± 0.162	0.061
Breast muscle				
Gln, μmol/mg prot	7.222^b^ ± 0.511	8.667^a^ ± 0.373	9.135^a^ ± 0.450	0.027
NH_3_, µg/mg prot	5.700^a^ ± 0.456	4.732^ab^ ±0.279	4.320^b^ ± 0.193	0.032
IGF-1, ng/mgprot	767.520^b^ ± 23.650	843.774^ab^ ±30.510	880.614^a^ ± 28.921	0.039
GDF-8, pg/mgprot	3,968.812^a^ ± 53.002	3,703.582^b^ ± 62.632	3,648.116^b^ ± 113.641	0.035
d 42				
Serum				
Gln, nmol/mL	311.968^b^ ± 26.232	353.701^ab^ ±24.465	424.094^a^ ± 31.009	0.039
NH_3_, μmol/mL	0.739^a^ ± 0.048	0.592^ab^ ±0.063	0.523^b^ ± 0.031	0.026
IGF-1, ng/mL	10.804^b^ ± 0.696	11.705^b^ ± 0.496	13.831^a^ ± 0.710	0.017
GH, ng/mL	2.464^b^ ± 0.036	2.455^b^ ± 0.072	2.677^a^ ± 0.038	0.016
Breast muscle				
Gln, μmol/mg prot	2.601^b^ ± 0.202	3.408^ab^ ±0.324	3.725^a^ ± 0.331	0.048
NH_3_, µg/mg prot	6.304 ± 0.289	6.285 ± 0.477	4.936 ± 0.528	0.080
IGF-1, ng/mgprot	816.271 ± 19.344	879.813 ± 23.350	897.374 ± 29.200	0.083
GDF-8, pg/mgprot	3,860.146^a^ ± 59.544	3,268.225^b^ ± 190.099	3,289.961^b^ ± 214.895	0.048

^a,b^ The data in the same row with shoulder labels containing different letters indicate significant differences (*P* < 0.05)

#### Expression analysis of nitrogen metabolism-related genes and pathways

As presented in Table 8, compared to the basal diet, OA supplementation significantly up-regulated the relative mRNA expression of *OAT* and *IGF-1* in the liver at d 21, as well as *HPRT*, *S6K1*, and *mTOR* at d 42 (*P* < 0.05). Furthermore, both the OA group and the PC group exhibited significantly higher hepatic mRNA levels of *HPRT* at d 21 and *IGF-1* at d 42 compared to the negative control (*P* < 0.05). Notably, both treatments also significantly up-regulated the expression of *mTOR* in the liver at d 21 (*P* < 0.05).

**Table 8:** Effects of dietary supplementation with an ornithine-aspartic acid mixture and OA on the expression of nitrogen metabolism-related genes and pathways in broiler chickens (*n* = 5)

Item	NC	PC	OA	*P*-value
d 21				
Liver				
*OAT*	1.000^b^ ± 0.023	1.145^ab^ ±0.064	1.241^a^ ± 0.057	0.019
*GS*	1.000 ± 0.067	1.190 ± 0.082	1.249 ± 0.065	0.072
*GDH*	1.000 ± 0.078	0.875 ± 0.072	0.840 ± 0.105	0.412
*XOD*	1.000 ± 0.077	0.811 ± 0.010	0.848 ± 0.046	0.056
*HPRT*	1.000^b^ ± 0.085	1.292^a^ ± 0.072	1.310^a^ ± 0.050	0.015
*IGF-1*	1.000^b^ ± 0.051	1.170^ab^ ±0.067	1.257^a^ ± 0.065	0.034
*S6K1*	1.000 ± 0.069	1.120 ± 0.054	1.251 ± 0.075	0.060
*Eif4E*	1.000 ± 0.045	1.084 ± 0.075	1.155 ± 0.084	0.327
*mTOR*	1.000^b^ ± 0.037	1.191^a^ ± 0.061	1.288^a^ ± 0.071	0.013
Breast muscle				
*GS*	1.000^b^ ± 0.070	1.176^ab^ ±0.065	1.243^a^ ± 0.050	0.045
*GDH*	1.000 ± 0.066	0.896 ± 0.158	0.862 ± 0.058	0.633
*XOD*	1.000^a^ ± 0.069	0.761^b^ ± 0.099	0.717^b^ ± 0.042	0.040
*HPRT*	1.000^b^ ± 0.075	1.174^ab^ ±0.023	1.318^a^ ± 0.100	0.031
*IGF-1*	1.000^b^ ± 0.050	1.187^ab^ ±0.094	1.357^a^ ± 0.070	0.017
*S6K1*	1.000^b^ ± 0.030	1.115^ab^ ±0.035	1.164^a^ ± 0.053	0.038
*Eif4E*	1.000 ± 0.045	1.110 ± 0.064	1.132 ± 0.071	0.296
*mTOR*	1.000^b^ ± 0.060	1.126^ab^ ±0.047	1.209^a^ ± 0.016	0.019
*GDF-8*	1.000^a^ ± 0.027	0.811^b^ ± 0.068	0.709^b^ ± 0.065	0.010
*NF-κB*	1.000^a^ ± 0.053	0.792^b^ ± 0.057	0.740^b^ ± 0.065	0.020
d 42				
Liver				
*OAT*	1.000 ± 0.068	1.154 ± 0.071	1.205 ± 0.043	0.091
*GS*	1.000 ± 0.053	1.145 ± 0.080	1.260 ± 0.086	0.084
*GDH*	1.000 ± 0.037	0.908 ± 0.112	0.903 ± 0.066	0.622
*XOD*	1.000 ± 0.033	0.845 ± 0.076	0.797 ± 0.048	0.056
*HPRT*	1.000^b^ ± 0.062	1.128^ab^ ±0.088	1.313^a^ ± 0.087	0.050
*IGF-1*	1.000^b^ ± 0.030	1.234^a^ ± 0.103	1.225^a^ ± 0.034	0.042
*S6K1*	1.000^b^ ± 0.085	1.115^ab^ ±0.037	1.323^a^ ± 0.079	0.021
*Eif4E*	1.000 ± 0.056	1.163 ± 0.093	1.159 ± 0.093	0.316
*mTOR*	1.000^b^ ± 0.017	1.128^ab^ ±0.085	1.268^a^ ± 0.043	0.018
Breast muscle				
*GS*	1.000^b^ ± 0.042	1.165^a^ ± 0.060	1.216^a^ ± 0.054	0.032
*GDH*	1.000 ± 0.049	0.859 ± 0.098	1.013 ± 0.079	0.337
*XOD*	1.000 ± 0.045	0.865 ± 0.070	0.782 ± 0.103	0.169
*HPRT*	1.000 ± 0.195	0.903 ± 0.055	1.096 ± 0.076	0.566
*IGF-1*	1.000^b^ ± 0.067	1.128^ab^ ±0.025	1.255^a^ ± 0.076	0.035
*S6K1*	1.000^b^ ± 0.050	1.238^a^ ± 0.054	1.174^a^ ± 0.057	0.023
*Eif4E*	1.000^b^ ± 0.064	1.175^a^ ± 0.034	1.264^a^ ± 0.062	0.016
*mTOR*	1.000^b^ ± 0.052	1.166^a^ ± 0.045	1.141^a^ ± 0.034	0.045
*GDF-8*	1.000 ± 0.052	0.836 ± 0.055	0.806 ± 0.069	0.081
*NF-κB*	1.000^a^ ± 0.086	0.652^b^ ± 0.057	0.747^b^ ± 0.068	0.013

^a,b^ The data in the same row with shoulder labels containing different letters indicate significant differences (*P* < 0.05)

The relative mRNA expression of nitrogen metabolism-related factors in breast muscle is shown in Table 8. At 21 days of age, dietary supplementation with 400 mg/kg OA significantly up-regulated the expression of nitrogen-metabolism-related genes, including *IGF-1*, *GS*, *mTOR*, and *S6K1*, compared to the negative control group (*P* < 0.05). Conversely, the supplemented groups had significantly down-regulated expression levels of *NF-κB*, *XOD* and *GDF-8*, and up-regulated expression of nitrogen metabolism-related genes such as *GS*, *S6K1*, and the *mTOR* signaling pathway compared to the basal diet control (*P* < 0.05).

### Exp. 2

#### Intestinal histology

The results from Exp. 1 indicate that OA supplementation exhibits a superior effect in broilers compared to the addition of a mixture of its constituent AAs. We hypothesized that this finding may be attributed to the differential absorption, utilization, and regulatory impact of OA on intestinal function. Therefore, this study further investigated intestinal histology to elucidate the potential mechanisms underlying the advantageous effects of OA. As shown in Table 9, compared with the negative control group, supplementation with 400 mg/kg OA significantly reduced duodenal CD in 21-day-old broilers (*P* < 0.05). Furthermore, compared with the basal diet, all supplementation treatments significantly increased the V/C in both jejunum (*P* < 0.05) and duodenum (*P* < 0.05), indicating a significant improvement in intestinal histology.

**Table 9:** Effects of dietary supplementation with an ornithine-aspartic acid mixture and OA on intestinal morphology in broiler chickens at d 21 (*n* = 5)

Item	NC	PC	OA	*P*-value
Duodenum				
VH, μm	1,340.054 ± 58.233	1,493.136 ± 32.855	1,436.688 ± 15.782	0.052
CD, μm	175.782^a^ ± 6.225	164.520^ab^ ±4.221	156.044^b^ ± 2.655	0.032
V/C	7.692^b^ ± 0.545	9.088^a^ ± 0.177	9.214^a^ ± 0.141	0.015
Jejunum				
VH, μm	996.462 ± 33.272	1,017.636 ± 19.580	1,086.286 ± 45.392	0.197
CD, μm	147.164 ± 1.864	133.406 ± 2.817	137.728 ± 6.344	0.095
V/C	6.784^b^ ± 0.296	7.632^a^ ± 0.100	7.900^a^ ± 0.159	0.006

^a,b^ The data in the same row with shoulder labels containing different letters indicate significant differences (*P* < 0.05)

#### AIAAD

As shown in Table 10, compared with the control group, dietary supplementation with 400 mg/kg OA significantly increased the AIAAD of aspartic acid, arginine, and Gln in broilers at d 21 (*P* < 0.05). Furthermore, OA supplementation resulted in a significant improvement in the digestibility of isoleucine, asparagine, and tryptophan (*P* < 0.01). These findings indicate that OA effectively enhances the digestion and utilization of dietary AAs.

**Table 10:** Effects of dietary supplementation with an ornithine-aspartic acid mixture and OA on AIAAD in broiler chickens at d 21 (*n* = 5), %

Item	NC	PC	OA	*P*-value
Histidine	75.528 ± 2.292	76.231 ± 2.191	77.015 ± 4.805	0.951
Isoleucine	69.667^b^ ± 3.926	72.390^b^ ± 1.804	83.640^a^ ± 1.462	0.006
Valine	86.145 ± 1.163	87.576 ± 1.040	87.651 ± 0.643	0.489
Asparagine	84.470^b^ ± 2.001	87.780^b^ ± 1.357	92.872^a^ ± 0.814	0.006
Threonine	86.647 ± 1.942	87.333 ± 1.327	89.576 ± 1.381	0.415
Tryptophan	83.778^b^ ± 0.573	85.180^b^ ± 0.808	88.128^a^ ± 0.924	0.006
Aspartic acid	77.252^b^ ± 0.369	80.296^ab^ ±2.012	83.838^a^ ± 0.716	0.010
Proline	74.269 ± 5.007	82.998 ± 2.453	86.005 ± 1.908	0.076
Lysine	90.191 ± 1.498	89.886 ± 1.028	88.939 ± 1.356	0.784
Arginine	88.455^b^ ± 1.201	90.607^ab^ ±0.893	92.594^a^ ± 0.873	0.039
Methionine	86.741 ± 2.147	87.176 ± 1.101	91.597 ± 0.747	0.068
Citrulline	79.088 ± 2.395	83.801 ± 3.093	80.466 ± 2.584	0.471
Gln	80.986^b^ ± 1.683	85.980^ab^ ±1.675	88.537^a^ ± 1.786	0.026
Glutamic acid	86.154 ± 2.008	87.009 ± 2.321	87.268 ± 1.389	0.915
Alanine	88.123 ± 1.501	90.047 ± 1.316	89.618 ± 1.233	0.588
Glycine	49.593 ± 10.639	53.478 ± 10.276	67.203 ± 3.077	0.357

^a,b^ The data in the same row with shoulder labels containing different letters indicate significant differences (*P* < 0.05)

#### Targeted metabolomics analysis of AAs

##### Screening of differential metabolites

**Fig. 1:**
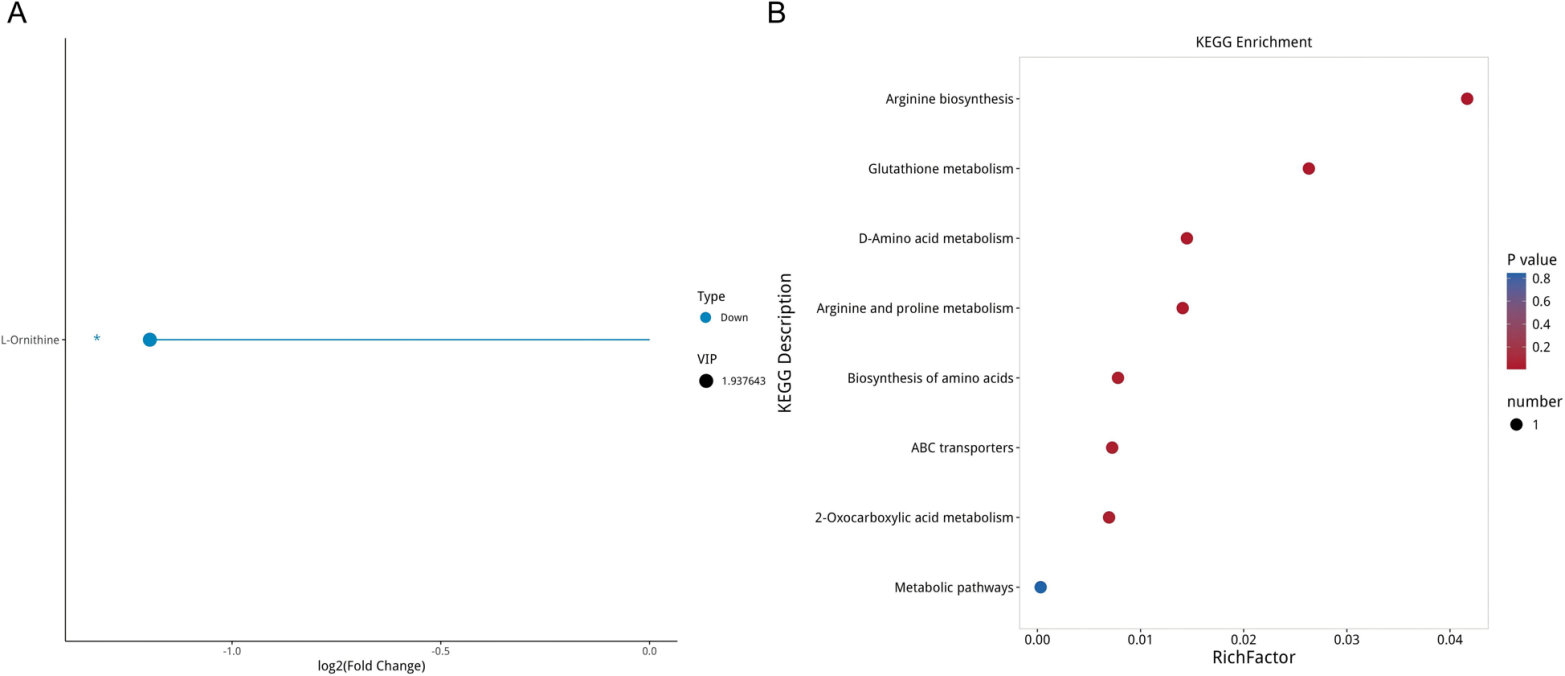
Effects of dietary supplementation with an ornithine-aspartic acid mixture and OA on the metabolomic profiles in the ileal chyme of broiler chickens at d 21 (*n* = 5). **A** Differential metabolites. **B** KEGG pathways and biological functions

As illustrated in Fig. 1A, metabolomic analysis identified L-ornithine as a key differential metabolite between the PC group and the OA group. Specifically, compared to the PC group, the OA group exhibited a significant down-regulation of L-ornithine levels in the ileal chyme of broilers at d 21 (*P* < 0.05). This reduction in ileal L-ornithine concentration suggests a potential enhancement in the absorption and utilization of ornithine in OA-supplemented broilers.

##### KEGG pathway analysis

As shown in Fig. 1B, KEGG pathway enrichment analysis revealed that, compared to the PC group, OA supplementation significantly affected AA metabolism pathways in the ileal chyme of 21-day-old broilers. The primary pathways affected included arginine biosynthesis (gga00220) and 2-Oxocarboxylic acid metabolism (gga01210). Specifically, the abundance of L-ornithine (C00077), a key metabolite enriched within these pathways, was significantly reduced in the ileal chyme of the OA group compared to the PC group (*P* < 0.05). These findings suggest that, potentially superior to a simple mixture of ornithine and aspartic acid, OA supplementation exerts its beneficial effects by facilitating the enhanced absorption and utilization of ornithine in broilers (Table 11).

**Table 11:** KEGG pathway enrichment analysis based on AA-targeted metabolomics (*n* = 5)

Pathway ID	Pathway	Level 1	Level 2	Down_meta	Up_meta	*P*-value
gga00220	Arginine biosynthesis	Metabolism	AA metabolism	L-Ornithine (Biosynthesis of AA|C00077)	-	0.006
gga00480	Glutathione metabolism	Metabolism	Metabolism of other AA	L-Ornithine (Biosynthesis of AA|C00077)	-	0.010
gga00470	D-AA metabolism	Metabolism	Metabolism of other AA	L-Ornithine (Biosynthesis of AA|C00077)	-	0.018
gga00330	Arginine and proline metabolism	Metabolism	AA metabolism	L-Ornithine (Biosynthesis of AA|C00077)	-	0.019
gga01230	Biosynthesis of AA	Metabolism	Global and overview maps	L-Ornithine (Biosynthesis of AA|C00077)	-	0.034
gga02010	ABC transporters	Environmental Information Processing	Membrane transport	L-Ornithine (Biosynthesis of AA|C00077)	-	0.037
gga01210	2-Oxocarboxylic acid metabolism	Metabolism	Global and overview maps	L-Ornithine (Biosynthesis of AA|C00077)	-	0.038
gga01100	Metabolic pathways	Metabolism	Global and overview maps	L-Ornithine (Biosynthesis of AA|C00077)	-	0.844

“Up_meta” and “Down_meta” indicate the number of metabolites enriched in the pathway that are up-regulated or down-regulated, respectively

#### Expression analysis of intestinal AA transporter-related genes

As presented in Table 12, dietary supplementation with 400 mg/kg OA significantly modulated the mRNA expression of AA transporters in the jejunum of 21-day-old broilers. Specifically, compared with the basal diet group, the OA-supplemented group exhibited significantly up-regulated mRNA expression levels of *SLC1A5*, *SLC7A6*, *SLC25A15*, and *SLC38A2* (*P* < 0.05). The supplemented groups showed significant down-regulation of the relative expression of *SLC1A1* and up-regulation of *SLC6A19* expression compared with the basal diet group (*P* < 0.05).

**Table 12:** Effects of dietary supplementation with an ornithine-aspartic acid mixture and OA on the mRNA expression of AA transporters in the jejunum of broiler chickens at d 21 (*n* = 5)

Item	NC	PC	OA	*P*-value
*SLC1A1*	1.000^a^ ± 0.047	0.758^b^ ± 0.069	0.709^b^ ± 0.094	0.032
*SLC1A4*	1.000 ± 0.041	1.054 ± 0.048	1.189 ± 0.068	0.071
*SLC1A5*	1.000^b^ ± 0.052	1.059^ab^ ±0.063	1.223^a^ ± 0.059	0.047
*SLC3A2*	1.000 ± 0.063	1.108 ± 0.057	1.078 ± 0.059	0.443
*SLC6A14*	1.000 ± 0.078	0.908 ± 0.055	0.953 ± 0.070	0.643
*SLC6A19*	1.000^b^ ± 0.055	1.206^a^ ± 0.067	1.242^a^ ± 0.038	0.018
*SLC7A5*	1.000 ± 0.026	1.111 ± 0.019	1.203 ± 0.094	0.080
*SLC7A6*	1.000^b^ ± 0.042	1.053^ab^ ±0.025	1.235^a^ ± 0.094	0.045
*SLC7A7*	1.000 ± 0.052	1.133 ± 0.105	1.207 ± 0.085	0.246
*SLC7A11*	1.000 ± 0.047	1.091 ± 0.042	1.087 ± 0.024	0.213
*SLC25A15*	1.000^b^ ± 0.027	1.156^ab^ ±0.060	1.297^a^ ± 0.078	0.013
*SLC38A1*	1.000 ± 0.049	1.165 ± 0.097	1.134 ± 0.045	0.229
*SLC38A2*	1.000^b^ ± 0.020	1.164^ab^ ±0.056	1.265^a^ ± 0.080	0.021

^a,b^ The data in the same row with shoulder labels containing different letters indicate significant differences (*P* < 0.05)

#### 16S rRNA sequencing of cecal microbiota

##### Species composition analysis

**Fig. 2:**
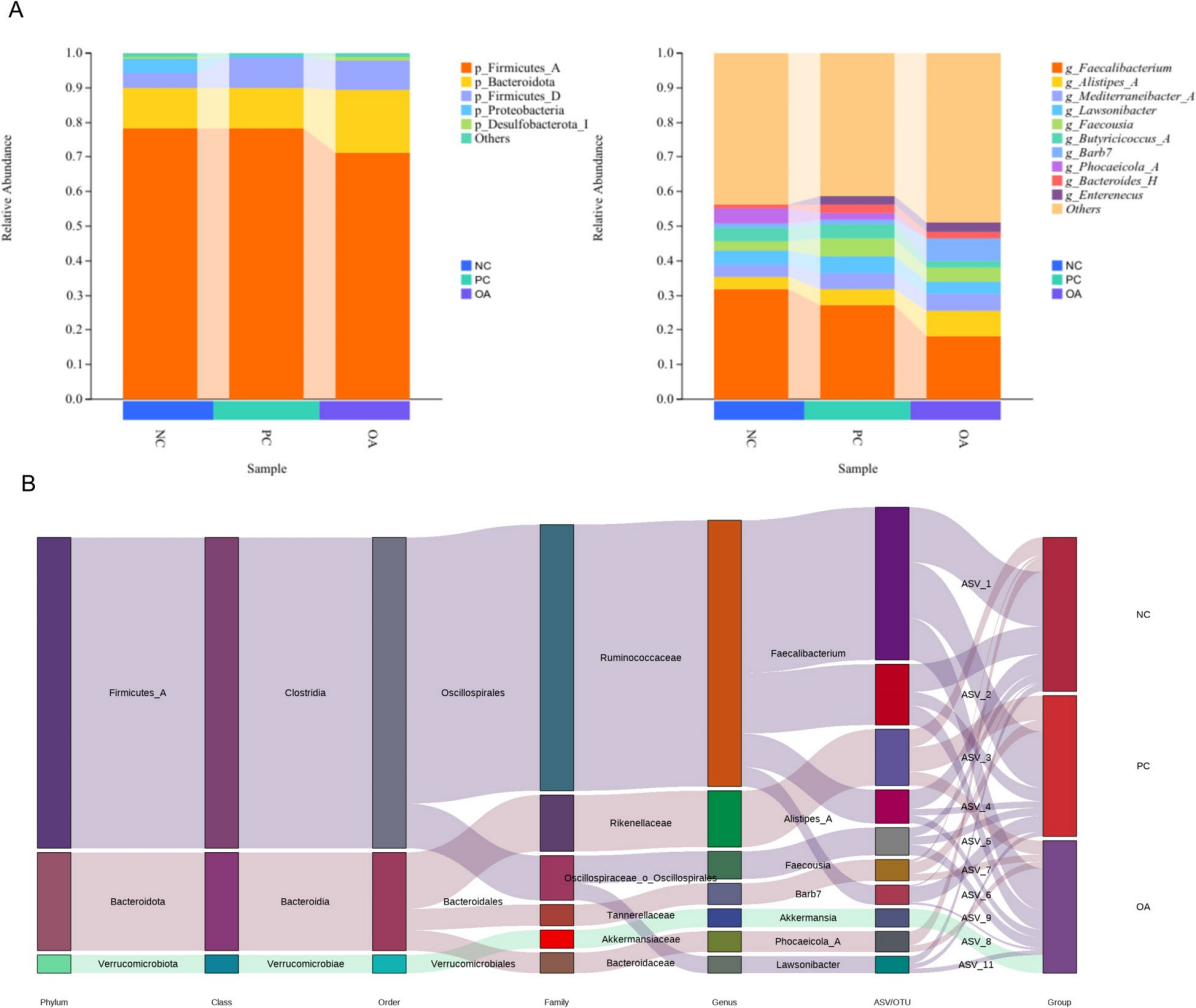
Effects of dietary supplementation with an ornithine-aspartic acid mixture and OA on the cecal microbiota of broiler chickens at d 21 (*n* = 5). **A** Relative abundance of cecal microbiota. **B** Cecal microbial composition

The taxonomic composition of the ileal microbiota at 21 days of age is presented in Fig. 2A. At the phylum level, the microbial community was dominated by Firmicutes_A, Bacteroidota, Firmicutes_D, Proteobacteria, and Desulfobacterota_I, which collectively represented the top five phyla in terms of relative abundance. At the genus level, the ten most abundant genera were identified as *Faecalibacterium*, *Alistipes_A*, *Mediterraneibacter_A*, *Lawsonibacter*, *Faecousia*, *Butyricicoccus_A*, *Barb7*, *Phocaeicola_A*, *Bacteroides_H*, and *Enterenecus*. Across all treatment groups, Firmicutes_A and Bacteroidetes were identified as the core phyla, while *Faecalibacterium* emerged as a key functional genus. Compared to the control group, the OA-supplemented group exhibited a significant enrichment in the relative abundance of *Akkermansia*. At the ASV/OTU level, ASV_1 and ASV_2, which were predominantly annotated to *Faecalibacterium*, exhibited higher relative abundance in the NC group. This suggests that the microbial community structure of the NC group was characterized by a “butyrate-producing bacteria-dominated” functional pattern. Conversely, in the PC group, an increase in the relative abundance of ASV_3 was observed, potentially linked to upstream regulation by *Bacteroidetes*. This implies that ornithine-aspartic acid mixture supplementation may improve gut microbiota composition and metabolism by influencing specific taxa such as *Bacteroidetes*. Furthermore, OA supplementation increased the flow width associated with ASV_9 and ASV_11 compared to the control group, corresponding to an elevated relative abundance of *Akkermansia*. These findings suggest that OA may enhance gut microbial homeostasis in 21-day-old broilers by promoting the proliferation of beneficial bacteria such as *Akkermansia* (Fig. 2B).

##### Diversity analysis

**Fig. 3:**
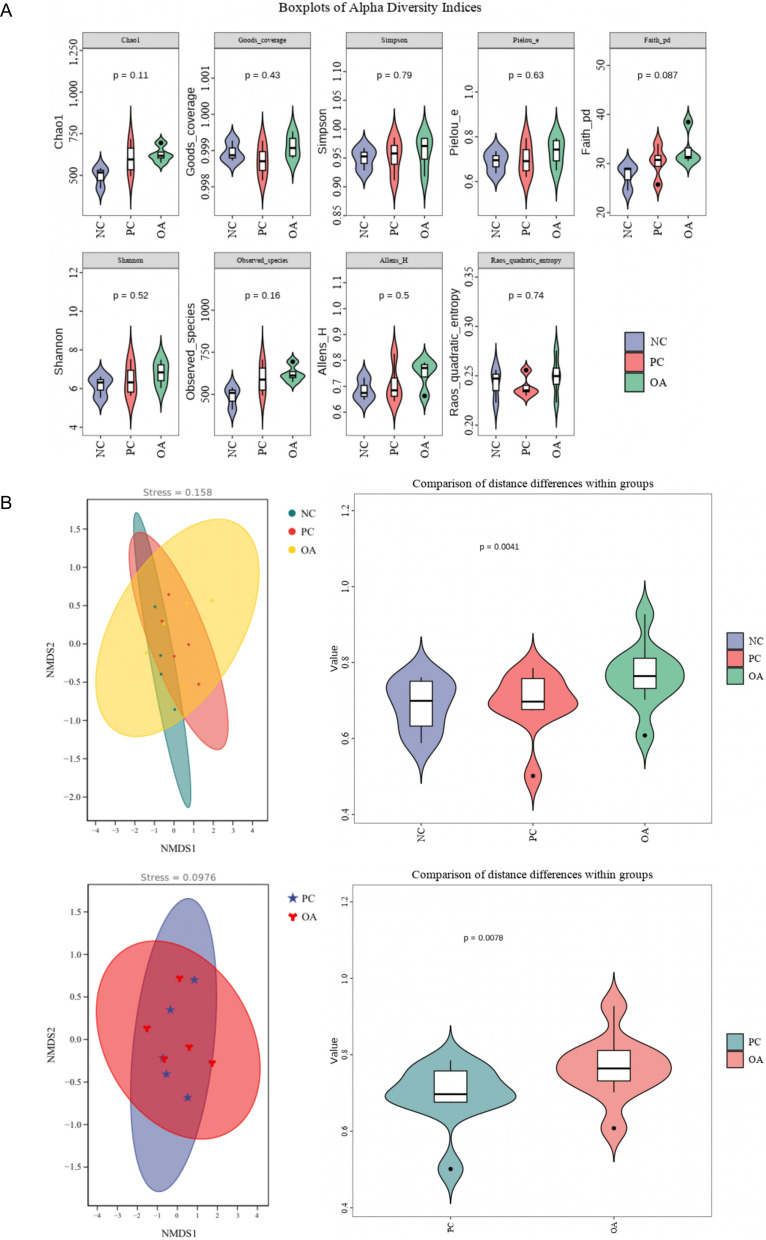
Effects of dietary supplementation with an ornithine-aspartic acid mixture and OA on the alpha and beta diversity of the cecal microbiota in broiler chickens at d 21 (*n* = 5). **A** Alpha diversity indices. **B** Beta diversity indices

Regarding alpha diversity, as illustrated in Fig. 3A, the OA group exhibited a relative trend toward a higher Faith PD index compared to the basal diet group at d 21 (*P* = 0.087), suggesting that dietary OA supplementation may enhance the diversity of the cecal microbiota in broilers. Furthermore, the OA group displayed enhanced microbial diversity compared to the group supplemented with a mixture of ornithine and aspartic acid. Beta diversity analysis revealed significant differences in microbial community structure. As shown in Fig. 3B, the microbial community structure of the OA group was significantly different from both the negative and positive controls. Specifically, the OA group showed a significant increase in the beta diversity of microbial communities in the cecal chyme at d 21 (*P* < 0.01). Furthermore, the OA group exhibited a significant separation from the PC group (*P* < 0.01).

##### Species differentiation and marker analysis

**Fig. 4:**
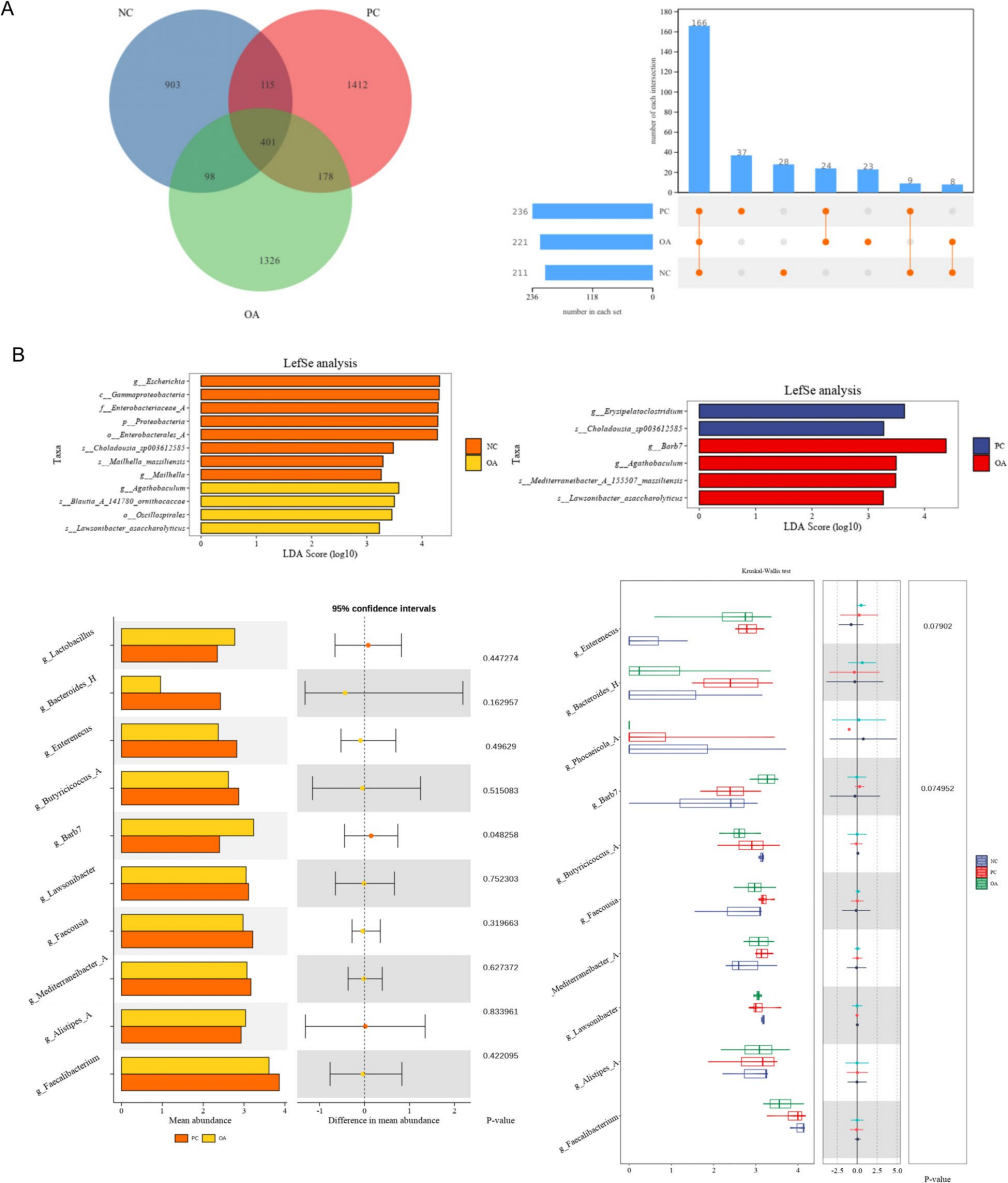
Effects of dietary supplementation with an ornithine-aspartic acid mixture and OA on the composition and relative abundance of cecal microbiota in broiler chickens at d 21 (*n* = 5). **A** Microbiota composition at the ASV/OTU and genus levels. **B** Relative abundance of microbial taxa

As shown in Fig. 4A, Venn diagram analysis at the ASV/OTU level revealed 401 shared OTUs among the NC, PC, and OA groups in the cecal chyme of 21-day-old broilers. The NC and PC groups exhibited 903 and 1,412 unique OTUs, respectively, while the OA supplementation group possessed 1,326 unique OTUs. At the genus level, 166 genera were common across all treatment groups. The NC and PC groups contained 28 and 37 unique genera, respectively, whereas the OA group contained 23 unique genera. Regarding specific bacterial abundance (Fig. 4B), all supplemented groups showed a tendency to increase the relative abundance of *Enterenecus* in the cecal chyme compared to the basal diet (*P* = 0.079). Furthermore, OA supplementation significantly increased the relative abundance of *Barb7* compared to the PC group (*P* < 0.05).

Linear discriminant analysis effect size analysis revealed that OA supplementation significantly modulated the cecal microbiota composition compared to the basal diet control group. Specifically, OA treatment reduced the relative abundance of *Escherichia* and Proteobacteria, while increasing the abundance of *Agathobaculum* and *Blautia_A_141780_ornithocaccae*. Furthermore, *Erysipelatoclostridium* and *Choladousia_sp003612585* were identified as discriminant taxa following the addition of the AA mixtures. Further analysis demonstrated that dietary supplementation with 400 mg/kg OA significantly enriched *Lawsonibacter_asaccharolyticus*, *Mediterraneibacter_A_155507_massiliensis*, and *Agathobaculum* at d 21 compared to the control group.

##### Microbial association network analysis

**Fig. 5:**
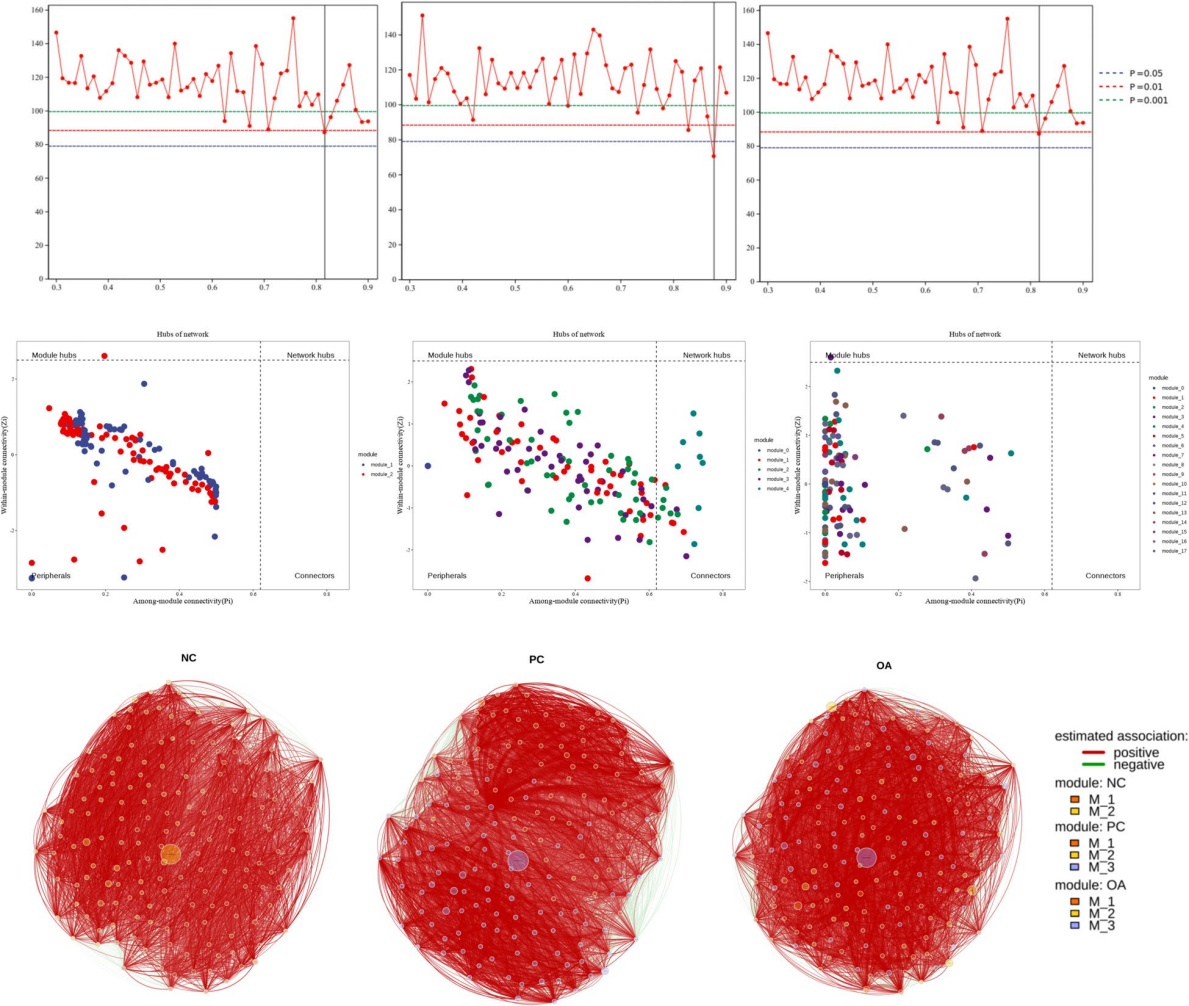
Effects of dietary supplementation with an ornithine-aspartic acid mixture and OA on genus-level microbial networks in broiler chickens at d 21 (*n* = 5)

**Fig. 6:**
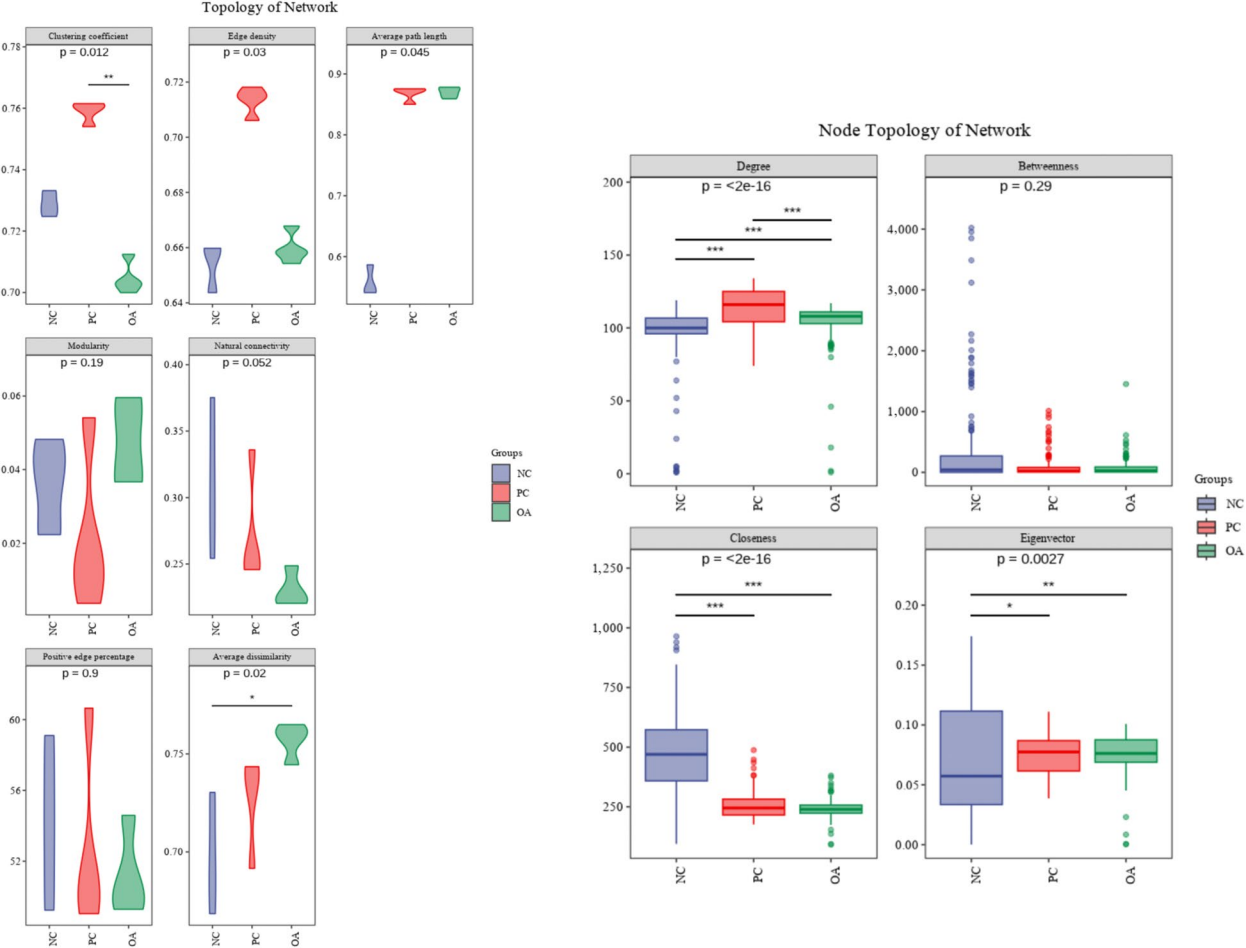
Effects of dietary supplementation with an ornithine-aspartic acid mixture and OA on the topological indices of microbial association networks at the genus level in broiler chickens at d 21 (*n* = 5)

As shown in Fig. 5, in the NC group, *Faecalibacterium*, *Alistipes_A*, and *Barb7* were identified as connectors, while *Butyricicoccus_A* served as a module hub. In the PC group, *Butyricicoccus_A*, *Anaerotruncus*, and *Anaerobutyricum* functioned as connectors. Conversely, in the OA group, *Alistipes_A* was identified as a module hub, whereas *Mediterraneiibacter_A*, *Streptococcus*, and *Anaerotruncus* acted as connectors. Additionally, compared to the basal diet group, both supplemented groups showed a significant increase in the average path length and a reduction in network efficiency, suggesting a distinct alteration in microbial interaction patterns compared to the basal dietary conditions. Furthermore, the OA group showed significantly increased average dissimilarity, indicating enhanced microbial community diversity. Notably, compared to the mixture group, OA treatment significantly reduced the clustering coefficient (*P* < 0.05), potentially promoting interactions among microorganisms of different functional groups. In other words, compared to the ornithine and aspartic acid mixture group, OA supplementation appeared to enhance interactions within microbial communities, potentially strengthening inter-microbial communication signals (Fig. 6). Moreover, compared to the basal diet group, all supplemented groups showed significantly increased Degree and Eigenvector indices, with a significant decrease in the Closeness index (*P* < 0.01). These shifts highlight the increased prominence and potential functional influence of core microbial taxa within the network. Collectively, these findings indicate that OA supplementation significantly enhances direct interactions and frequency within the microbial community, thereby facilitating the potential transfer of biological information between microbial populations (Fig. 6).

##### Predicted functional analysis of the cecal microbiota

**Fig. 7:**
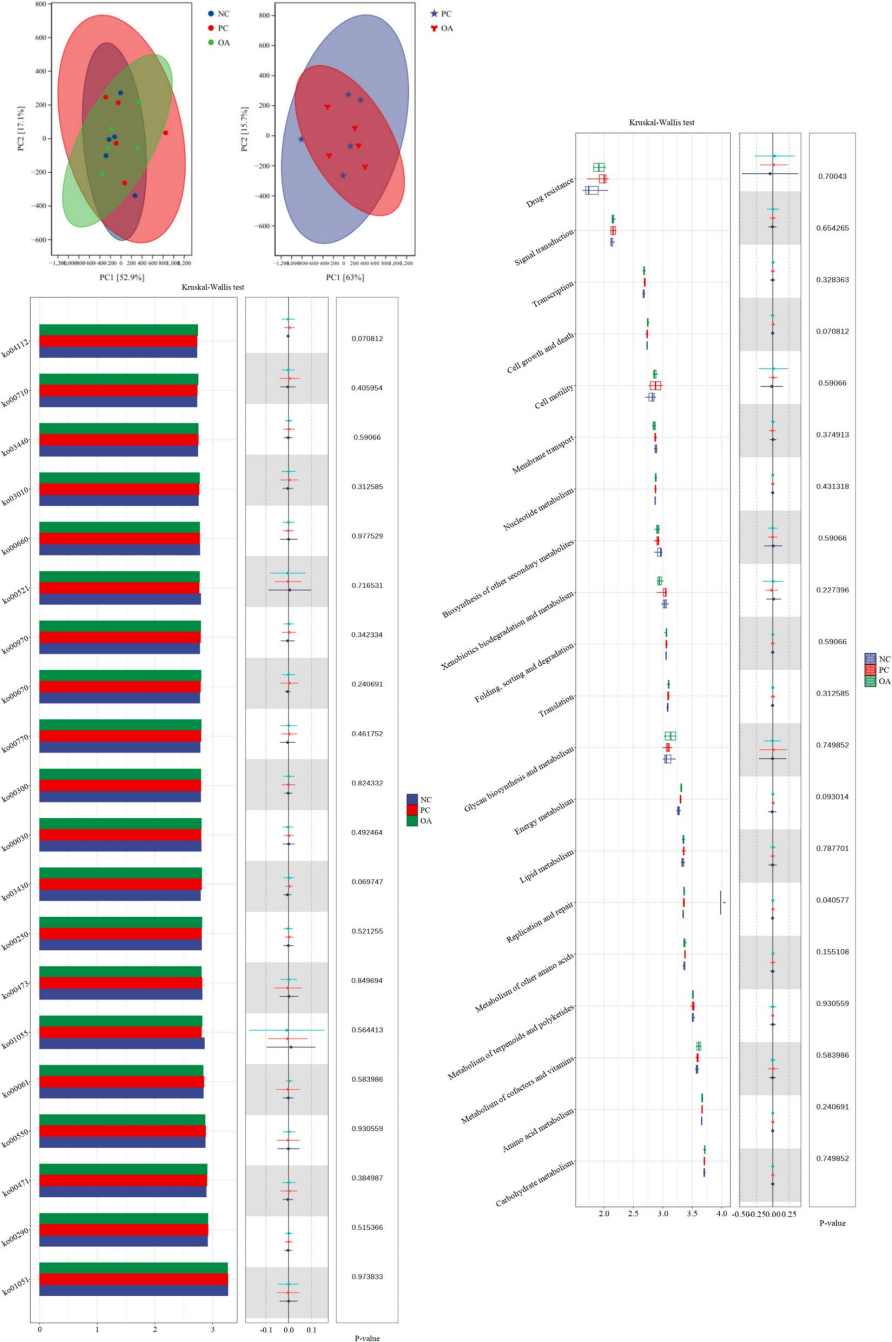
Effects of dietary supplementation with an ornithine-aspartic acid mixture and OA on microbial functional differences in broiler chickens at d 21 (*n* = 5)

To further elucidate the impact of OA on microbial metabolic functions, the PC and OA groups were selected for functional prediction analysis. Further differential analysis of the above functional pathways (Fig. 7) revealed distinct alterations in the cecal microbiota of 21-day-old broilers. Compared with the basal diet, the PC group exhibited a tendency toward enrichment of the mismatch repair pathway (ko03430; *P* = 0.070), while the OA group showed a relatively increasing trend in the cell cycle pathway (ko04112; *P* = 0.071). At KEGG Level 2, the replication and repair pathway was significantly up-regulated in all supplemented groups compared with the basal diet (*P* < 0.05). Furthermore, the OA group displayed a tendency toward up-regulation in the cell growth and death pathway (*P* = 0.071) and microbial energy metabolism pathways (*P* = 0.093) compared with the control group.

##### Microbial species contributions to metabolic pathways

**Fig. 8:**
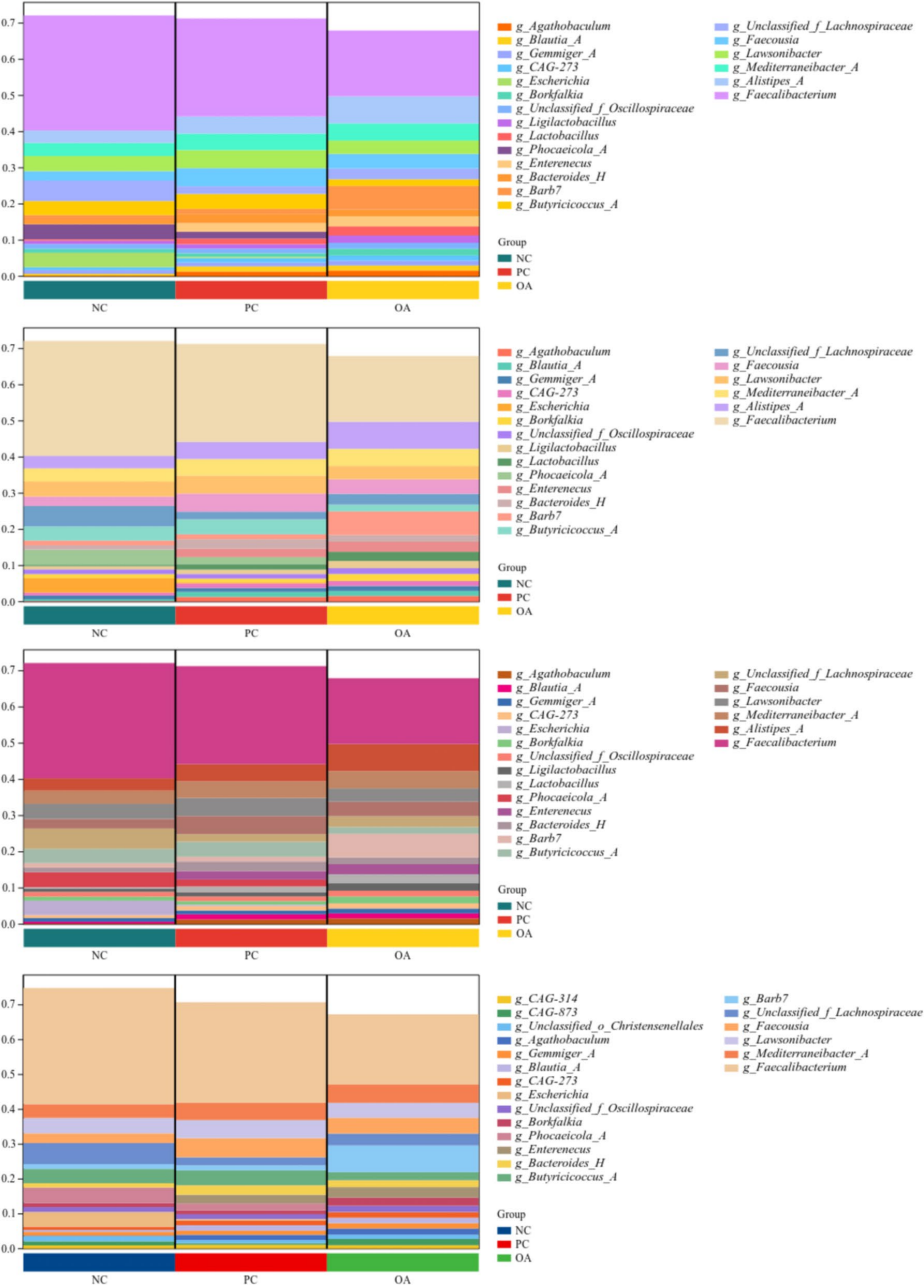
Effects of dietary supplementation with an ornithine-aspartic acid mixture and OA on the contribution of microbial species in the cecum of broiler chickens at d 21 (*n* = 5)

As shown in Fig. 8, species contribution analysis was performed at the genus level, focusing on AA metabolism pathways, specifically alanine, aspartate, and glutamate metabolism (KO00250) and metabolism of other AAs. In the PC group, the top five contributing taxa were *Faecalibacterium*, *Faecousia*, *Lawsonibacter*, *Alistipes_A*, and *Mediterraneibacter_A*. Following OA supplementation, the top five microbial species contributing to the cecal chyme of 21-day-old broilers shifted to *Faecalibacterium*, *Alistipes_A*, *Barb7*, *Mediterraneibacter_A*, and *Faecousia*. Furthermore, analysis based on the insulin signaling pathway (KO04910) revealed distinct alterations in the microbial contributors within all supplemented groups. Notably, compared to the PC group, OA supplementation significantly increased the specific contribution of *Barb7* to this pathway.

##### Feature analysis

**Fig. 9:**
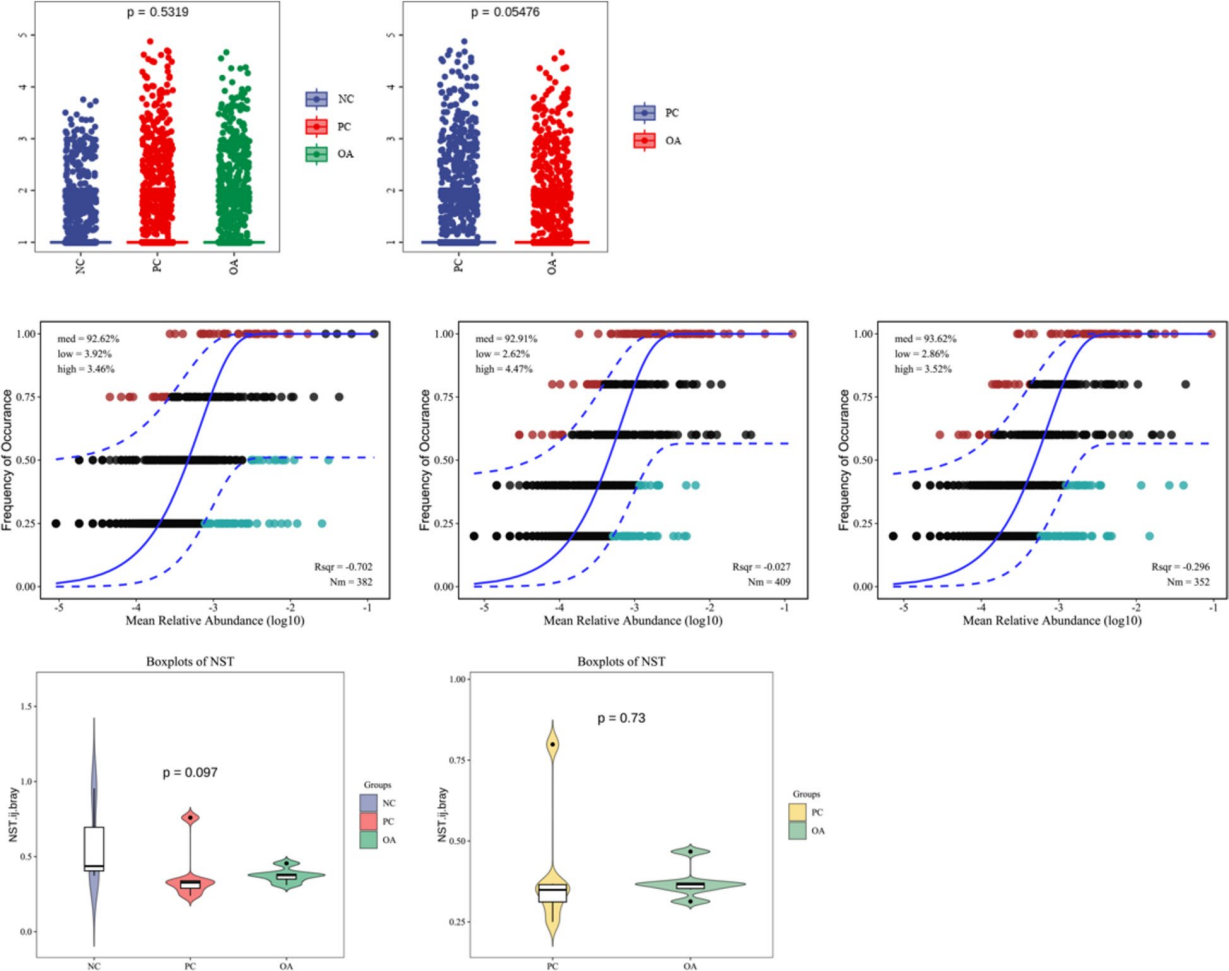
Effects of dietary supplementation with an ornithine-aspartic acid mixture and OA on microbial characteristic indices in broiler chickens at d 21 (*n* = 5)

As illustrated in Fig. 9, dietary supplementation with 400 mg/kg OA exhibited a tendency toward a reduced species niche breadth index compared to the positive control group (*P* = 0.055). This observation suggests that OA may enhance the environmental adaptability of the gut microbiota, thereby promoting the maintenance of species diversity. To further elucidate the mechanisms underlying community assembly, the NCM and NST analyses were performed. Compared to the basal diet, all supplemented groups showed an increased Rsqr index, indicating an improved goodness-of-fit of the neutral community model. This suggests that during community assembly, the microbial communities in the supplemented groups were increasingly driven by stochastic processes rather than deterministic processes. Furthermore, compared to both positive and negative control groups, the OA group reduced the Nm index and decreased the correlation between occurrence frequency and regional relative abundance. Concurrently, NST analysis indicated a tendency for all supplemented groups to reduce the NST index of the cecal microbiota in 21-day-old broilers compared to the basal diet (*P* = 0.097).

#### Concentrations of SCFAs in cecal chyme

As presented in Table 13, dietary supplementation with 400 mg/kg OA significantly increased the concentration of acetic acid in the cecal chyme of 21-day-old broilers compared with the basal diet group (*P* < 0.05). Both the OA supplementation group and the PC group exhibited significantly higher concentrations of propionic acid compared with the basal diet group (*P* < 0.05). Furthermore, the OA supplementation resulted in a significant elevation in butyric acid concentration compared with both the positive and negative control groups (*P* < 0.05).

**Table 13:** Effects of dietary supplementation with an ornithine-aspartic acid mixture and OA on the concentrations of cecal SCFAs in broiler chickens at d 21 (*n* = 5), µmol/g

Item	NC	PC	OA	*P*-value
Acetic acid	18.559^b^ ± 0.867	21.400^ab^ ±2.288	26.032^a^ ± 2.069	0.042
Propionic acid	3.023^b^ ± 0.157	4.151^a^ ± 0.333	4.567^a^ ± 0.457	0.020
Butyric acid	2.085^b^ ± 0.184	2.247^b^ ± 0.272	2.976^a^ ± 0.171	0.027
Valeric acid	0.236 ± 0.030	0.356 ± 0.118	0.337 ± 0.096	0.604
Isobutyric acid	0.314 ± 0.053	0.505 ± 0.077	0.462 ± 0.074	0.161
Isovaleric acid	0.317 ± 0.037	0.393 ± 0.034	0.437 ± 0.092	0.394

^a,b^ The data in the same row with shoulder labels containing different letters indicate significant differences (*P* < 0.05)

### Correlation analysis

#### Correlation analysis between growth performance and intestinal histology

**Fig. 10:**
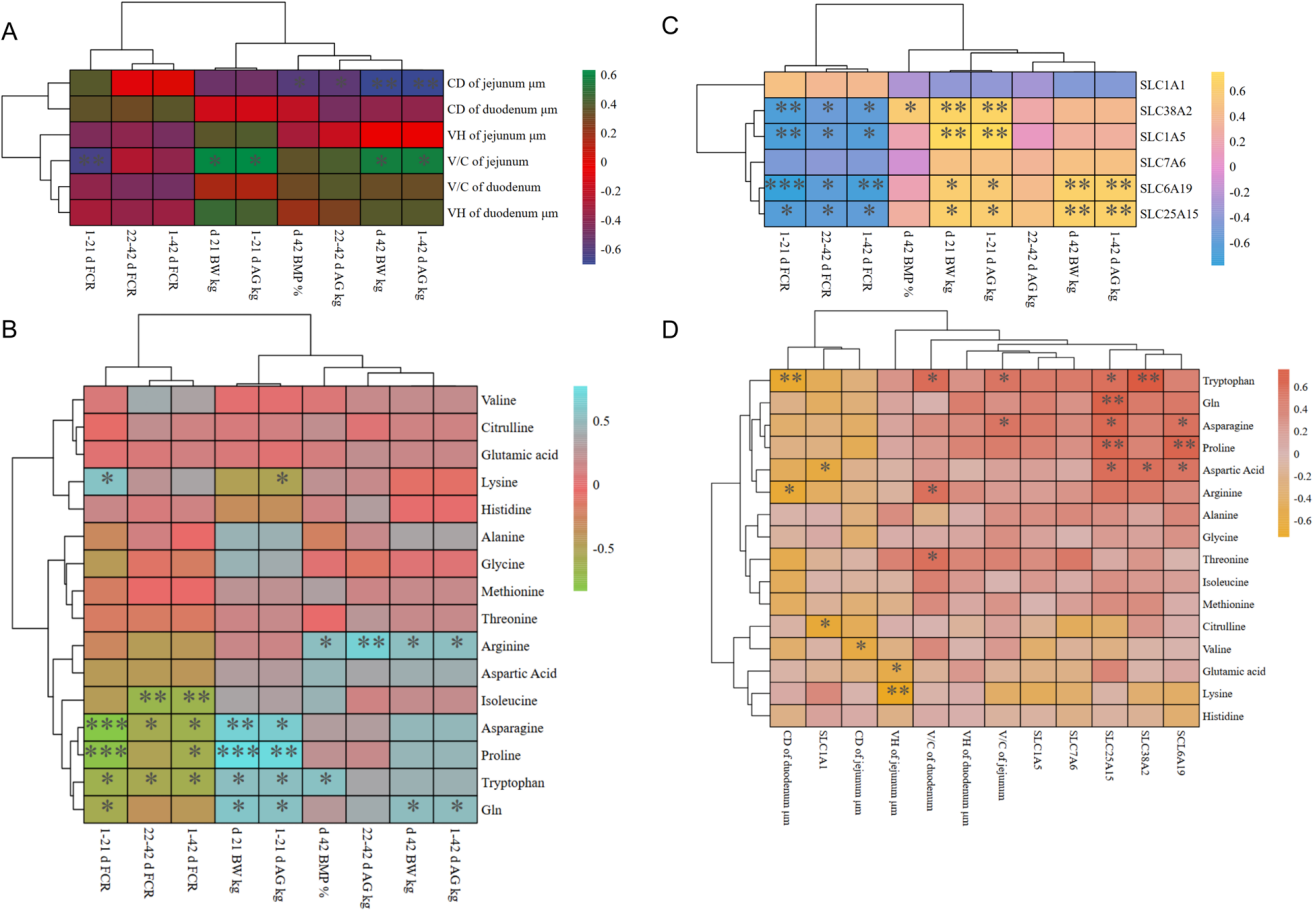
Effects of dietary supplementation with an ornithine-aspartic acid mixture and OA on the correlations among growth performance, intestinal morphology, AIAAD, and jejunal AA transporter gene expression (*n* = 5). **A** Correlation between growth performance and intestinal morphology. **B** Correlation between growth performance and AIAAD. **C** Correlation between growth performance and jejunal AA transporter gene expression. **D** Correlation between AIAAD, intestinal morphology, and jejunal AA transporter gene expression

As shown in Fig. 10A, jejunal CD exhibited a highly significant negative correlation with BW at d 42 and AG from d 1 to 42 (*P* < 0.01). Conversely, the jejunal V/C was negatively correlated with FCR from d 1 to 21 (*P* < 0.01). Furthermore, jejunal CD at d 21 showed a significant negative correlation with AG from d 22 to 42 and BMP at d 42 (*P* < 0.05). The V/C ratio of the jejunum at d 21 was positively correlated with BW at d 21 and AG from d 1 to 21 (*P* < 0.05). Collectively, these findings demonstrate a strong association between jejunal morphological integrity in 21-day-old broiler chickens and growth performance, suggesting that improvements in jejunal histomorphology are closely linked to enhanced growth performance in broilers.

#### Correlation analysis between growth performance and AIAAD

As shown in Fig. 10B, the AIAAD of specific AAs at d 21, notably proline and asparagine, showed a highly significant positive correlation with BW at d 21 and a significant negative correlation with FCR from d 1 to 21 (*P* < 0.01). Furthermore, the AIAAD of Gln and arginine was positively correlated with BW at d 42 and AG from d 1 to 42 (*P* < 0.05). Collectively, these results indicate that the AIAAD at d 21 is closely associated with growth performance, suggesting that enhanced AA digestibility contributes to improved growth performance and feed efficiency in broilers.

#### Correlation analysis between growth performance and jejunal AA transporter gene expression

As illustrated in Fig. 10C, the mRNA expression levels of AA transporter factors, specifically *SLC6A19* and *SLC25A15*, exhibited significant positive correlations with BW at d 42 and AG from d 1 to 42 (*P* < 0.01). These genes also showed significant positive correlations with BW at d 21 and AG from d 1 to 21 (*P* < 0.05). Additionally, the expression of jejunal AA transporters *SLC1A5* and *SLC38A2* at d 21 showed a highly significant negative correlation with FCR from d 1 to 21 (*P* < 0.01), as well as with FCR from d 22 to 42 (*P* < 0.05).

#### Correlation analysis between AIAAD and intestinal morphology and jejunal AA transporter gene expression

As shown in Fig. 10D, the AIAAD of specific AAs, such as tryptophan, was positively correlated with the mRNA expression levels of jejunal AA transporters, including *SLC38A2* and *SLC25A15*. Conversely, tryptophan digestibility showed a significant negative correlation with duodenal CD (*P* < 0.01) and a positive correlation with the V/C in both the duodenum and jejunum (*P* < 0.05). Furthermore, the expression of *SLC25A15* in the jejunum was positively correlated with the AIAAD of Gln and proline (*P* < 0.01), as well as aspartic acid (*P* < 0.05). These results indicate a close association among intestinal morphology, the expression of jejunal AA transporters, and AIAAD. Collectively, these findings suggest that improvements in intestinal structural integrity may facilitate AA transport across the gut epithelium, thereby enhancing AIAAD and promoting the efficient absorption and utilization of AAs in broilers.

In summary, dietary supplementation with OA improved the histological morphology of duodenal and jejunal epithelium, enhanced the AIAAD, and up-regulated the mRNA expression of AA transporters in the jejunum. These physiological alterations facilitated the absorption and utilization of dietary AAs, thereby improving growth performance and breast muscle development. Collectively, these findings elucidate the mechanism by which OA exerts its beneficial effects on the intestinal function of broilers.

#### Correlation analysis between cecal dominant microbial communities and indicators of intestinal AA absorption and utilization

**Fig. 11:**
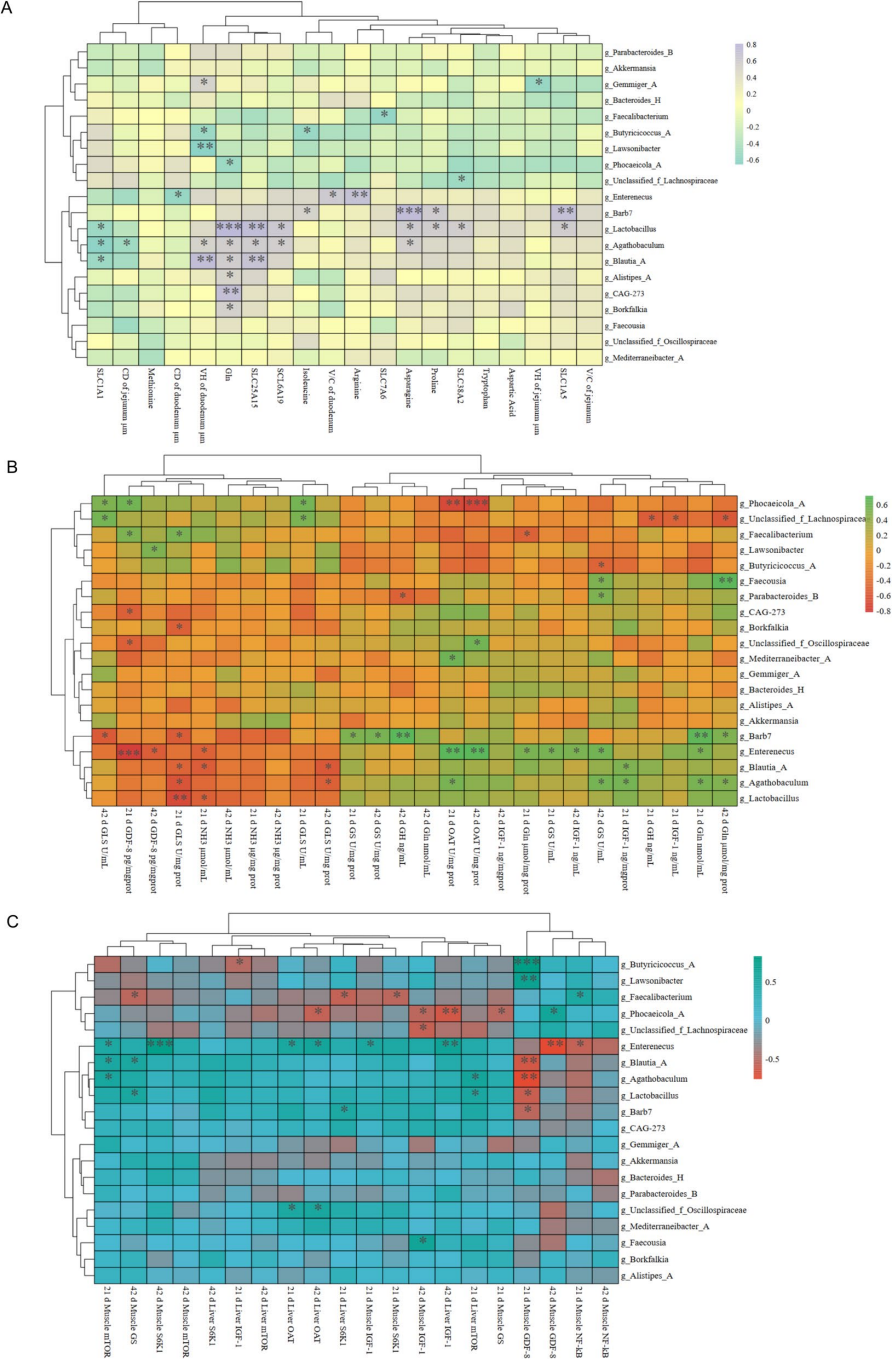
Effects of dietary supplementation with an ornithine-aspartic acid mixture and OA on the correlations between cecal dominant microbial communities and host metabolic parameters (*n* = 5). **A** Correlation with indicators of intestinal AA absorption and utilization. **B** Correlation with nitrogen metabolism indices. **C** Correlation with nitrogen metabolism-related gene expression

To elucidate the potential mechanisms by which OA modulates the gut microbiota distinct from a simple mixture of ornithine and aspartic acid, a correlation analysis was performed between the relative abundance of dominant cecal microbial taxa at d 21 and key indicators of AA absorption and utilization. As shown in Fig. 11A, the relative abundance of *Enterobacter* was significantly positively correlated with arginine digestibility (*P* < 0.01) and the V/C in the duodenum (*P* < 0.05), while being negatively correlated with CD (*P* < 0.05). Notably, the abundance of beneficial genera, such as *Lactobacillus*, exhibited a highly significant positive correlation with the AIAAD of Gln (*P* < 0.01). Furthermore, *Lactobacillus* abundance was positively associated with the mRNA expression of *SLC25A15* (*P* < 0.01) and *SLC38A2* (*P* < 0.05) in the jejunum, but negatively correlated with *SLC1A1* expression (*P* < 0.05). Additionally, gut core microbiota, such as *Blautia_A*, showed positive correlations with duodenal VH, Gln digestibility, and the relative expression of *SLC25A15* in the jejunum, while exhibiting a negative correlation with *SLC1A1* (*P* < 0.05). These findings indicate the intimate link between gut microbiota composition and AA absorption and utilization. Specifically, dietary OA supplementation enriched the abundance of beneficial bacteria, including *Lactobacillus* and *Blautia_A*, which in turn appeared to enhance the absorption and utilization of dietary AAs, thereby maximizing the beneficial effects of OA.

#### Correlation analysis between cecal dominant microbial communities and nitrogen metabolism indices

To elucidate the potential mechanisms underlying the beneficial effects of OA, a correlation analysis was conducted between the cecal dominant microbial communities and key indicators of Gln metabolism and protein synthesis from Exp. 1 (Fig. 11B). The relative abundance of *Phocaeicola_A* exhibited a highly significant negative correlation with hepatic OAT levels (*P* < 0.01), while showing significant positive correlations with serum GLS levels and GDF-8 content in the breast muscle at d 21 (*P* < 0.05). *Faecalibacterium* abundance was negatively correlated with breast muscle Gln content but positively correlated with GDF-8 and GLS levels in the breast muscle of 21-day-old broilers (*P* < 0.05). Furthermore, hepatic OAT activity displayed a highly significant positive correlation with the relative abundance of *Enterenecus* (*P* < 0.01). OAT activity at d 21 was positively correlated with specific genera such as *Mediterraneiibacter_A* and *Agathobaculum* (*P* < 0.05). *Blautia_A*, a gut core bacterium, showed a significant positive correlation with breast muscle IGF-1 levels, whereas it was negatively correlated with GLS activity and blood ammonia levels at d 21 (*P* < 0.05). Notably, *Lactobacillus* showed negative correlations with blood ammonia levels at d 21 (*P* < 0.05) and breast muscle GLS activity (*P* < 0.01).

As illustrated in Fig. 11C, correlation analysis revealed significant associations between specific cecal bacterial genera and gene expression. The abundance of *Lactobacillus* in the cecum of 21-day-old broilers was negatively correlated with *GDF-8* expression in breast muscle, while exhibiting a significant positive correlation with hepatic *mTOR* expression at d 21 and breast muscle *GS* expression at d 42 (*P* < 0.05). *Blautia_A* exhibited a highly significant negative correlation with breast muscle *GDF-8* expression at d 21 (*P* < 0.01), and a significant positive correlation with hepatic *mTOR* expression at d 21 and breast muscle *GS* expression at d 42. Regarding hepatic protein synthesis, the expression levels of *IGF-1* and *mTOR* were negatively associated with *Phocaeicola_A* and *Faecalibacterium*, but significantly positively correlated with *Agathobaculum* (*P* < 0.05). In breast muscle, the expression of protein synthesis-related factors, including *IGF-1*, *S6K1*, and *mTOR*, showed significant negative correlations with *Phocaeicola_A* abundance, whereas positive correlations were observed with *Enterenecus* (*P* < 0.05). Furthermore, hepatic *OAT* expression showed a significant positive correlation with *g_Unclassified_f_Oscillospiraceae* and *Enterenecus*, and a negative correlation with *Phocaeicola_A* (*P* < 0.05).

Collectively, these findings suggest that dietary OA supplementation facilitates the absorption and utilization of nutrients, particularly AAs and proteins, by modulating the composition and metabolism of the gut microbiota. Furthermore, OA enhances Gln metabolism within both hepatic and breast muscle tissues, which promotes Gln synthesis and enhances NH_3_ detoxification capacity, ultimately driving protein synthesis and deposition, thereby improving growth performance. Notably, compared to a simple mixture of ornithine and aspartic acid, OA induced distinct alterations in the cecal microbiota, specifically regarding the abundance of *Agathobaculum* and *Enterobacteriaceae*. These specific microbial shifts may represent a key factor contributing to the superior beneficial effects observed with OA supplementation.

## Discussion

Growth performance and slaughter performance serve as fundamental indicators for evaluating the efficacy of dietary interventions in poultry production. In the present study, compared to the basal diet group, all supplemented groups exhibited a significant reduction in FCR from d 1 to 21 and an increase in BW at d 21, which improved growth performance. Furthermore, supplementation with 400 mg/kg OA significantly increased BW at d 21 and AG from d 1 to 21, while consistently reducing FCR from d 22 to 42 and d 1 to 42. OA supplementation demonstrated superior efficacy in improving growth performance compared to the mixture group. Additionally, all supplemented groups showed a significant increase in BMP at d 42 compared to the negative control group. This elevation in relative breast muscle weight suggests that OA positively modulates muscle accretion and promotes overall development in broilers. It is worth noting that while OA supplementation significantly improved AG and FCR, no significant difference was observed in AFI among the treatment groups. This lack of effect on feed intake aligns with our previous findings and suggests that the growth-promoting mechanism of OA is not mediated through appetite stimulation. Instead, the improved growth performance in the absence of increased feed consumption may indicate a marked enhancement in nutrient utilization efficiency.

Serum biochemical parameters serve as critical biomarkers for evaluating the physiological health and reflecting the nutritional metabolic status of broilers [20]. Specifically, ALT and AST are pivotal indicators of hepatic metabolism, where elevated activities of these enzymes are classically associated with hepatocellular injury [21]. In the present study, dietary supplementation with OA significantly reduced serum ALT and AST activities at d 21 compared to the basal diet, suggesting a protective effect against hepatic injury. Furthermore, supplementation with OA or the ornithine-aspartic acid mixture significantly increased serum TP levels at d 21, while concurrently reducing UA concentrations. Notably, this reduction in serum UA persisted in the OA group at d 42. Serum TP and UA are established indices of nitrogen metabolism, reflecting the dynamics of protein synthesis and AA utilization efficiency [22, 23]. Consequently, the observed elevation in TP combined with the reduction in UA suggests that OA enhances protein synthesis and optimizes AA utilization. Collectively, these findings indicate that dietary supplementation with OA and an ornithine-aspartic acid mixture modulates nitrogen metabolism to favor protein anabolism and AA utilization, thereby effectively mitigating liver damage and promoting muscle development in broilers.

The liver serves as a key organ for nitrogen metabolism in broilers, playing a pivotal role in determining growth performance [24]. Disruption of hepatic nitrogen homeostasis inevitably compromises growth, leading to reduced BW and increased morbidity [25]. Therefore, this study focused on evaluating key indicators of hepatic nitrogen metabolism in broilers. It has been reported that OA facilitates NH_3_ detoxification and Gln synthesis by enhancing GS activity while reducing GLS activity, thereby promoting overall growth and health [26–28]. In the present study, compared to the basal diet, all supplemented groups significantly increased serum GS activity at d 42, elevated Gln levels, and reduced blood ammonia concentrations at d 42, effectively mitigating systemic NH_3_ accumulation. Furthermore, the OA group exhibited a more pronounced effect than the group receiving a simple mixture of ornithine and aspartic acid. Specifically, OA supplementation significantly increased hepatic OAT activity and decreased serum GLS activity at d 42, leading to higher Gln content and lower blood ammonia levels. These findings align with our previous observations, suggesting that OA, as an ionic salt, may offer superior efficacy compared to its free AA counterparts. Mechanistically, our results revealed that both supplemented groups promoted protein anabolism by activating the hepatic *mTOR* signaling pathway. Furthermore, compared to the basal diet, the OA group significantly up-regulated the mRNA expression of *OAT* in the livers of 21-day-old broilers, which correlated with the observed increase in hepatic OAT activity. This enhancement in OAT function facilitates the transamination of ornithine. Therefore, it is reasonable to infer that the superior efficacy of OA over the AA mixture may be attributed to the enhanced transamination efficiency of ornithine, potentially driven by the unique physicochemical properties of the OA salt form.

IGF-1, primarily synthesized by the liver, acts as a pivotal metabolic regulator which influences the growth and development of the body [29]. In the present study, OA supplementation significantly elevated serum IGF-1 concentrations in 21-day-old broilers and up-regulated hepatic *IGF-1* expression compared to the negative control group. Furthermore, at 42 days of age, the addition of 400 mg/kg OA in the diet significantly increased serum IGF-1 and GH levels compared to the control groups. These hormonal elevations were consistent with the observed increase in BW.

Dietary AAs undergo transamination and deamination reactions to generate metabolic precursors that regulate biological pathways essential for the growth and metabolism of broilers [6]. Notably, NH_3_ production is inextricably tied to Gln metabolism [30, 31]. Beyond functioning as a byproduct of protein catabolism, NH_3_ acts as a critical mediator within the liver-muscle axis. Hepatic dysfunction can precipitate hyperammonemia, driving excessive NH_3_ accumulation and uptake by muscle tissue. This influx disrupts systemic nitrogen balance, exacerbating liver injury and inducing muscle wasting, which ultimately compromises growth performance and health [32–34]. Given that muscle tissue is the primary extrahepatic site for NH_3_ detoxification via Gln synthesis, the observed improvements in BW and BMP in the present study suggest that all supplemented groups promote breast muscle development by enhancing protein synthesis and deposition. To elucidate the underlying mechanism, this study further analyzed nitrogen metabolism-related markers in breast muscle. Compared to the negative control group, all supplemented groups showed a significant reduction in GLS activity and an increase in Gln concentrations of breast muscle at d 21. Furthermore, dietary supplementation with 400 mg/kg OA significantly increased GS activity and reduced intramuscular NH_3_ accumulation. These results are similar to previous findings suggesting that OA mitigates the adverse effects of sarcopenia by enhancing Gln metabolism [26, 35, 36]. At the molecular level, all supplemented groups up-regulated the mRNA expression of *GS* and key anabolic signaling factors, including *S6K1* and *mTOR*, compared to the basal diet. This up-regulation provides molecular evidence for enhanced protein synthesis and deposition. Moreover, OA supplementation increased both the content and mRNA expression of *IGF-1* in breast muscle at d 21. Studies have shown that IGF-1 is a potent driver of muscle hypertrophy and development [37, 38], and the elevated IGF-1 levels provide a mechanistic explanation for the concomitant improvements in growth performance, such as increased BW, observed in this study.

In the present study, all supplemented groups exhibited a significant reduction in serum UA levels compared to the basal diet, implying that dietary supplementation with OA and an ornithine-aspartic acid mixture modulates purine metabolism in broilers. In broiler chickens, NH_3_ generated from nitrogen metabolism is partially converted into UA via the purine metabolic pathway for subsequent excretion [39, 40]. To elucidate this mechanism, this study also measured key enzymes involved in this pathway. Our results revealed that all supplemented groups significantly reduced XOD activity in the breast muscle of 21-day-old broilers and down-regulated *XOD* expression. XOD serves as a rate-limiting enzyme in purine catabolism; its inhibition leads to a reduction in purine breakdown and a consequent decrease in UA synthesis [6]. Therefore, the OA-induced suppression of purine catabolism observed in this study provides a plausible mechanistic explanation for the concurrent decrease in serum UA concentrations.

Furthermore, the present study demonstrated that dietary supplementation with OA, as well as a mixture of ornithine and aspartic acid, significantly reduced GDF-8 content and down-regulated the relative mRNA expression of *NF-κB* and *GDF-8* in breast muscle at d 21, compared to the negative control. GDF-8, also known as myostatin, functions as a potent negative regulator of skeletal muscle growth and development [41]. Numerous studies have demonstrated that excessive intramuscular NH_3_ accumulation triggers GDF-8 production via activation of the NF-κB signaling pathway, thereby inhibiting muscle growth and development [26, 42, 43]. Similar to these observations, our findings suggest that OA may mitigate muscle wasting by attenuating the NH_3_-induced, *NF-κB*-mediated up-regulation of *GDF-8*. This is similar to previous reports positing that OA reduces the risk of sarcopenia through the suppression of GDF-8 levels [44].

Collectively, the present findings demonstrate that OA exhibits superior efficacy in regulating Gln metabolism, enhancing protein synthesis and deposition, and mitigating NH_3_ accumulation compared to a simple mixture of ornithine and aspartic acid. This enhanced efficacy is primarily attributed to two mechanisms: the increase of hepatic OAT levels and the improved intestinal utilization efficiency of AAs, particularly ornithine. Biochemically, OAT catalyzes the conversion of ornithine and α-ketoglutarate into Glu, thereby providing the essential precursor for Gln synthesis [45]. Data from Exp. 1 reveal that dietary supplementation with 400 mg/kg OA significantly increased both the enzymatic activity and relative mRNA expression of *OAT* in the liver. This up-regulation facilitated the transamination of ornithine, ensured an ample substrate supply for Gln synthesis, and ultimately promoted growth and development. Consequently, these results validate the hypothesis that the modulation of hepatic OAT activity and expression represents a primary mechanism underlying the beneficial effects of OA in broilers.

The intestine constitutes the primary site for nutrient digestion and absorption in broilers, and its histomorphological architecture is intrinsically linked to growth performance and development. Consequently, intestinal absorptive capacity is frequently evaluated through microscopic structural analysis. Among the various parameters, intestinal VH, CD, and V/C serve as critical indices for assessing intestinal function [7]. In the present study, compared with the basal diet, all supplemented groups increased the V/C ratio in the duodenum and jejunum, which improved intestinal epithelial integrity. Furthermore, OA treatment reduced duodenal CD in broilers at 21 days of age, potentially enhancing intestinal absorptive efficiency. These findings are consistent with previous research [46], indicating that dietary supplementation with OA improves intestinal epithelial morphology, thereby potentially facilitating the absorption and utilization of nutrients, particularly AAs.

In the intestine, OA dissociates into ornithine and aspartic acid, which are subsequently absorbed and utilized by broilers primarily via active transport mechanisms [8]. These AAs enter intestinal epithelial cells through specific transporters, such as SLC25A15 and SLC1A5, and exert their biological effects upon binding to intracellular receptors [9, 10]. Notably, the complex salt structure of OA may confer distinct advantages over monomeric AAs [14]. In the present study, all supplemented groups showed an up-regulation of *SLC6A19* in the jejunum of broilers at d 21 and a down-regulation of *SLC1A1* compared to the basal diet. SLC6A19 is known to modulate gut microbiota balance and nutrient utilization efficiency by regulating the transport of neutral AAs, including methionine [47]. Furthermore, supplementation with 400 mg/kg OA significantly up-regulated the expression of *SLC1A5*, *SLC25A15*, and *SLC38A2* in the jejunum of 21-day-old broilers. SLC1A5, also known as ASCT2, is a neutral AA transporter with high affinity for Gln [48, 49], while SLC25A15 functions as a mitochondrial ornithine transporter, facilitating the translocation of ornithine across the inner mitochondrial membrane (a critical step in the urea cycle) [9]. These findings suggest that, compared to a mixture of ornithine and aspartic acid, OA supplementation effectively enhances the expression of key AA transporters. This up-regulation likely promotes the digestion, absorption, and transport of dietary AAs, particularly Gln and ornithine, thereby improving overall protein utilization efficiency in broilers.

The results from Exp. 1 indicate that dietary supplementation with an ornithine-aspartic acid mixture or OA modulates the liver-Gln-muscle axis, thereby influencing protein deposition and NH_3_ metabolism, which ultimately enhances the growth performance of broilers. Specifically, the therapeutic effects of OA appear to be mediated primarily by the metabolic actions of its constituent AAs, ornithine and aspartic acid. Furthermore, Exp. 2 demonstrated that OA supplementation improves the intestinal absorption and utilization efficiency of AAs. Therefore, to elucidate the underlying mechanisms and validate the hypothesis that OA enhances bioavailability, targeted AA metabolomics was performed on ileal chyme samples collected at d 21. Metabolomics analysis serves as a powerful tool for evaluating metabolic pathways and substrate transformation within biological systems [50]. In the present study, ornithine was identified as a significant differential metabolite between the PC and OA groups. Specifically, dietary supplementation with 400 mg/kg OA significantly down-regulated ornithine concentrations in the ileal chyme compared to the control group. This reduction in ileal ornithine abundance indicates enhanced intestinal absorption of ornithine derived from OA. KEGG pathway enrichment analysis further revealed that OA significantly influenced AA metabolism pathways, particularly gga00220. This alteration, driven by the significant reduction in ileal ornithine, aligns with the physiological improvements observed in Exp. 1. In summary, these findings indicate that OA exerts its beneficial effects by facilitating the efficient absorption and utilization of ornithine in broiler chickens.

The gut microbiota plays a fundamental role in nutrient metabolism and host defense, thereby regulating the physiological status of the host [51, 52]. Notably, the diversity of the microbiome is intrinsically linked to host health [53]. In the present study, OA supplementation exhibited a tendency to increase the Faith PD index in the cecal microbiota of 21-day-old broilers. Furthermore, compared to the PC group, OA supplementation altered the microbial community structure, increased beta diversity and enhanced species richness. Reduced microbial diversity is widely recognized as a key indicator of dysbiosis, whereas higher diversity supports a stable intestinal microenvironment and confers colonization resistance against pathogens [54]. LEfSe analysis revealed that, compared to the basal diet, all supplemented groups showed a trend toward increased relative abundance of *Enterenecus* in the cecal chyme of 21-day-old broilers. Conversely, OA treatment reduced the abundance of *Escherichia* and Proteobacteria, while enriching core beneficial taxa such as *Blautia_A_141780_ornithocaccae* and improving gut microbiota composition. An expansion of *Escherichia* and Proteobacteria is frequently associated with gut dysbiosis and compromised homeostasis [55–57]. *Blautia* is a gut symbiont known to promote intestinal health and function as a potential probiotic [58].

Furthermore, compared to the group supplemented with the ornithine and aspartic acid mixture, the 400 mg/kg OA treatment significantly increased the relative abundance of *Agathobaculum*, *Lawsonibacter_asaccharolyticus*, and *Barb7*, thereby improving the gut microbiota profile of the broiler chickens. Given that microbiota composition is a critical determinant of intestinal health [59], these shifts are physiologically significant. Both *Agathobaculum* and *Lawsonibacter_asaccharolyticus* are known to maintain microbial homeostasis by promoting the production of SCFAs [60–62]. Our findings are similar to previous clinical observations in cirrhotic patients, where OA improved health outcomes by modulating gut microbiota composition [44]. Collectively, the results indicate that, compared to the simple AA mixture, OA supplementation more effectively modulates the gut microbial structure. This is characterized by an enrichment of beneficial genera, such as *Blautia* and *Agathobaculum*, and a concomitant reduction in the colonization of potential pathogens like *Escherichia* and Proteobacteria, ultimately enhancing microbial homeostasis. Functionally, KEGG analysis revealed that OA supplementation was associated with an up-regulation of pathways related to microbial energy metabolism. Furthermore, network analysis is a tool used to evaluate ecological interactions among microbial communities [63]. It was reported that increased connectivity correlates with community complexity and stability, leading to an increase in microbial interactions [64]. In the present study, compared to the PC group, OA treatment promoted stronger inter-microbial interactions and signaling, establishing a more complex microbial network.

SCFAs, the primary fermentation products of the gut microbiota, play a pivotal role in maintaining intestinal microbial homeostasis [65]. In the present study, all supplemented groups exhibited significantly elevated concentrations of propionic acid. Furthermore, compared to the PC group, OA supplementation increased butyric acid levels in the cecal chyme of 21-day-old broilers. This elevation is consistent with the observed increase in the relative abundance of butyrate-producing bacteria, specifically *Agathobaculum*. Notably, our data suggest that the dietary addition of 400 mg/kg OA enhances the absorption and utilization of dietary AAs and protein, which is superior to a simple mixture of ornithine and aspartic acid. This effect appears to be mediated through the modulation of gut microbiota composition and metabolic function. Given the intrinsic link between intestinal health and growth, this study demonstrates that OA supplementation improves broiler performance through a coordinated mechanism: enhancing intestinal epithelial morphology, up-regulating the expression of AA transporters, and consequently increasing AA digestibility and utilization.

To elucidate the mechanisms underlying the beneficial effects of OA on the gut, this study first analyzed the correlations between dominant cecal microbial communities and indices of intestinal AA absorption and utilization. Our results indicated that the relative abundance of beneficial genera, such as *Blautia_A*, was positively correlated with markers of AA absorption and utilization. This suggests that OA may facilitate AA uptake and metabolism by modulating the structural composition of the gut microbiota. Furthermore, given the established link between the gut microbiome and muscle development in broilers [66, 67], specifically the influence of gut microbes on intramuscular protein synthesis and deposition [68], this study further conducted a correlation analysis between dominant cecal taxa and markers of Gln metabolism and protein anabolism. We observed that beneficial taxa, including *Agathobaculum* and *Blautia_A*, exhibited a negative correlation with NH_3_ levels, while demonstrating a positive correlation with the content and expression of key factors involved in Gln metabolism and protein anabolism, such as GS, IGF-1, and *mTOR*. These findings imply that OA supplementation enhances broiler growth performance by regulating the intestinal microbiota, which subsequently modulates Gln metabolism and protein synthesis in liver and breast muscle tissues. This is similar to previous reports indicating that SCFAs, such as butyric acid, regulate host growth by influencing muscle and liver function [69]. Moreover, OAT levels were found to be significantly correlated with *Agathobaculum* abundance. This suggests that, potentially distinct from a simple mixture of ornithine and aspartic acid, the specific efficacy of OA may be attributed to its ability to induce targeted alterations in the cecal microbiota, particularly the enrichment of *Agathobaculum*.

## Conclusion

Compared to a simple physical mixture of ornithine and aspartic acid, the supplementation of OA in a corn-SBM-based diet significantly enhances the intestinal absorption and utilization of AAs, particularly ornithine. This improvement effectively optimizes intestinal function, thereby maximizing the nutritional efficacy and beneficial effects of the supplement. Furthermore, OA modulates microbial metabolism by regulating the composition of the gut microbiota, notably characterized by an enrichment of *Agathobaculum* and a reduction in *Escherichia* abundance. Collectively, these mechanisms promote breast tissue development, ultimately improving growth performance of the broiler chickens.

## Supplementary Information

Additional file 1: Table S1. Oligonucleotide primer sequences used for real-time fluorescence quantitative PCR. Table S2. Oligonucleotide primer sequences used for real-time fluorescence quantitative PCR.

## Data Availability

The datasets used or analysed during the current study are available from the corresponding author on reasonable request.

## Abbreviations

AAs: Amino acids
AFI: Average feed intake
AFP: Abdominal fat percentage
AG: Average gain
AIAAD: Apparent ileal AA digestibility
ALB: Albumin
ALT: Alanine aminotransferase
ANOVA: One-way analysis of variance
AST: Aspartate aminotransferase
BMP: Breast muscle percentage
BUN: Blood urea nitrogen
CD: Crypt depth
CP: Crude protein
DP: Dressing percentage
EP: Eviscerated percentage
FCR: Feed conversion rate
GDF-8: Myostatin
GH: Growth hormone
GLB: Globulin
Gln: Glutamine
GLS: Glutaminase
Glu: Glutamate
GS: Glutamine synthase
HEP: Half-eviscerated percentage
HPRT1: Hypoxanthine phosphoribosyltransferase
IGF-1: Insulin-like growth factor 1
LC–MS/MS: Liquid chromatography-tandem mass spectrometry
LMP: Leg muscle percentage
NC: Negative control
NH_3_: Ammonia
OA: L-Ornithine-L-aspartate
OAT: Ornithine aminotransferase
PC: Positive control
QC: Quality control
SBM: Soybean meal
SCFAs: Short-chain fatty acids
SEM: Standard error of the mean
TIC: Total ion chromatograms
TiO_2_: Titanium dioxide
TP: Total protein
UA: Uric acid
V/C: VH to CD ratio
VH: Villi height
XOD: Xanthine oxidase
