# Supplementary material for: L-Ornithine-L-aspartate enhances growth performance and nitrogen metabolism via modulation of intestinal amino acid transporters and microbiota in broilers
Source: J Anim Sci Biotechnol. 2026 Jul 3;17:137. doi: 10.1186/s40104-026-01450-7 (PMC13330389; doi:10.1186/s40104-026-01450-7)
Supplement: Supplementary file 1 — Additional file 1: Table S1. Oligonucleotide primer sequences used for real-time fluorescence quantitative PCR. Table S2. Oligonucleotide primer sequences used for real-time fluorescence quantitative PCR. [file 40104_2026_1450_MOESM1_ESM.doc]

**Table S1** Oligonucleotide primer sequences used for real-time fluorescence quantitative PCR^a^

| **Genes^b^** | **Forward (5´→3´)** | **Reverse (5´→3´)** |
| --- | --- | --- |
| *β-actin* | CAACACAGTGCTGTCTGGTGGTAC | CTCCTGCTTGCTGATCCACATCTG |
| *GS* | GTGGAACTTTGATGGCTC | TATCCATAATCCGCCTACAG |
| *GDH* | CAATGCTCACAAGGTCAA | TTCAGCCATTCAAAGTAGGA |
| *XOD* | GGGGAAGATGGTGAGATGGA | ACGATGCGATTTGATGGGAC |
| *HPRT1* | CCCAAACATTATGCAGACGA | TGTCCTGTCCATGATGAGC |
| *IGF-1* | TGTACTGTGCTCCAATAAAGC | CTGTTTCCTGTGTTCCCTCTACTTG |
| *S6K1* | GGTGGAGTTTGGGGGCATTA | GAAGAACGGGTGAGCCTAA |
| *Eif4E* | TGGAACCGGAAACCACTCCC | GCGCCCATCTGTTTTGTAGTG |
| *mTOR* | AGTGAGAGTGATGCGGAGAG | GAAACCTTGGACAGCGGG |
| *OAT* | CGAGGGCTTTCTACAATGA | CTCCACCCCTGTATTCATAG |
| *GDF-8* | GGTATCTGGCAGAGTATTGATGTGAA | CAAAATCTCTGCGGGACCGT |
| *NF-κB* | GTG TGA AGA AAC GGG AAC TG | GGC ACG GTT GTC ATA GAT GG |

^a^ Primers were designed using Primer Express software (Sangon Biotech (Shanghai) Co., Ltd, Shanghai, China)

^b^ *GS* Glutathione synthetase, *GDH* Glutamate dehydrogenase, *XOD* Xanthine oxidase, *HPRT* Hypoxanthine phosphoribosyltransferase, *IGF-1* Insulin-like growth factor 1, *S6K1* Ribosomal protein S6 kinase 1, *Eif4E* Eukaryotic initiation factor 4E binding protein-1, *mTOR* Mammalian target of rapamycin, *OAT* Ornithine aminotransferase, *GDF-8* Growth differentiation factor-8, *NF-κB* Nuclear factor kappa-B

**Table S2** Oligonucleotide primer sequences used for real-time fluorescence quantitative PCR

| **Genes^a^** | **Forward (5´→3´)** | **Reverse (5´→3´)** |
| --- | --- | --- |
| *β-actin* | CAACACAGTGCTGTCTGGTGGTAC | CTCCTGCTTGCTGATCCACATCTG |
| *SLC1A1* | GGGCTCGTATTCTGATGGCA | CCACAAGCACTTGGCCTTTC |
| *SLC1A4* | TTGGCCGGGAAGGAGAAG | AGACCATAGTTGCCTCATTGAATG |
| *SLC1A5* | GATTGTGGAGATGGAGGATGTGG | TGCGAGTGAAGAGGAAGTAGATGA |
| *SLC3A2* | AGGTCCTGCTGAAGTTCTCC | CATCTTGCCACCAATCGAGG |
| *SLC6A14* | CTCCAGTGGGCTGGATGAGA | CAAGGCAGCTCCAACGATCA |
| *SLC6A19* | TATCCTGGCTGGGTCTATGC | AGGCCTGTACGATCCCTTCT |
| *SLC7A5* | GATTGCAACGGGTGATGTGA | CCCCACACCCACTTTTGTTT |
| *SLC7A6* | GCCCTGTCAGTAAATCAGACAAGA | TTCAGTTGCATTGTGTTTTGGTT |
| *SLC7A7* | GAAAACCTCAGAGCTCCCTT | GAGGTAAATTCCTCTCGGGG |
| *SLC7A11* | TGTGAGAGTCTGGGTGGAAC | AAATCTGAATGCGGGCACTC |
| *SLC25A15* | CCTGCTCTGGTAGCCAACAT | CGGCACTTCACCAGCTCT |
| *SLC38A1* | CGCTAAATGCAACATCACCTATC | TGGTGGGCAAAGCATACA |
| *SLC38A2* | GAACAAGTAGGGCCCTGTAATC | GGGCAGAGCTTGATGTTATCT |

^a^ *SLC1A1* Solute carrier family 1 member 1 gene, *SLC1A4* Solute carrier family 1 member 4 gene, *SLC1A5* Solute carrier family 1 member 5 gene, *SLC3A2* Solute carrier family 3 member 2 gene, *SLC6A14* Solute carrier family 6 member 14 gene, *SLC6A19* Solute carrier family 6 member 19 gene, *SLC7A5* Solute carrier family 7 member 5 gene, *SLC7A6* Solute carrier family 7 member 6 gene, *SLC7A7* Solute carrier family 7 member 7 gene, *SLC7A11* Solute carrier family 7 member 11 gene, *SLC25A15* Solute carrier family 25 member 15 gene, *SLC38A1* Solute carrier family 38 member 1 gene, *SLC38A2* Solute carrier family 38 member 2 gene
